# Therapeutic Effect of Repetitive Transcranial Magnetic Stimulation for Post-stroke Vascular Cognitive Impairment: A Prospective Pilot Study

**DOI:** 10.3389/fneur.2022.813597

**Published:** 2022-03-22

**Authors:** Byoungwoo Cha, Jongwook Kim, Jong Moon Kim, Joo-Wan Choi, Jeein Choi, Kakyeong Kim, Jiook Cha, MinYoung Kim

**Affiliations:** ^1^Department of Rehabilitation Medicine, CHA Bundang Medical Center, CHA University School of Medicine, Seongnam, South Korea; ^2^Rehabilitation and Regeneration Research Center, CHA University School of Medicine, Seongnam, South Korea; ^3^Department of Brain and Cognitive Sciences, College of Natural Sciences, Seoul National University, Seoul, South Korea; ^4^Department of Psychology, College of Social Sciences, Seoul National University, Seoul, South Korea; ^5^AI Institute, Seoul National University, Seoul, South Korea

**Keywords:** chronic stroke, post-stroke vascular cognitive impairment, depression, repetitive transcranial magnetic stimulation, ipsilesional dorsolateral prefrontal cortex

## Abstract

**Objective:**

Post-stroke cognitive impairment (PSCI) is resistant to treatment. Recent studies have widely applied repetitive transcranial magnetic stimulation (rTMS) to treat various brain dysfunctions, such as post-stroke syndromes. Nonetheless, a protocol for PSCI has not been established. Therefore, this study is aimed to evaluate the therapeutic effect of our high-frequency rTMS protocol for PSCI during the chronic phase of stroke.

**Methods:**

In this prospective study, ten patients with PSCI were enrolled and received high-frequency rTMS on the ipsilesional dorsolateral prefrontal cortex (DLPFC) for 10 sessions (5 days per week for 2 weeks). Cognitive and affective abilities were assessed at baseline and 2 and 14 weeks after rTMS initiation. To investigate the therapeutic mechanism of rTMS, the mRNA levels of pro-inflammatory cytokines (interleukin (IL)-6, IL-1β, transforming growth factor beta [TGF-β], and tumor necrosis factor alpha [TNF-α]) in peripheral blood samples were quantified using reverse transcription polymerase chain reaction, and cognitive functional magnetic resonance imaging (fMRI) was conducted at baseline and 14 weeks in two randomly selected patients after rTMS treatment.

**Results:**

The scores of several cognitive evaluations, i.e., the Intelligence Quotient (IQ) of Wechsler Adult Intelligence Scale, auditory verbal learning test (AVLT), and complex figure copy test (CFT), were increased after completion of the rTMS session. After 3 months, these improvements were sustained, and scores on the Mini-Mental Status Examination and Montreal Cognitive Assessment (MoCA) were also increased (*p* < 0.05). While the Geriatric Depression Scale (GeDS) did not show change among all patients, those with moderate-to-severe depression showed amelioration of the score, with marginal significance. Expression of pro-inflammatory cytokines was decreased immediately after the ten treatment sessions, among which, IL-1β remained at a lower level after 3 months. Furthermore, strong correlations between the decrease in IL-6 and increments in AVLT (*r* = 0.928) and CFT (*r* = 0.886) were found immediately after the rTMS treatment (*p* < 0.05). Follow-up fMRI revealed significant activation in several brain regions, such as the medial frontal lobe, hippocampus, and angular area.

**Conclusions:**

High-frequency rTMS on the ipsilesional DLPFC may exert immediate efficacy on cognition with the anti-inflammatory response and changes in brain network in PSCI, lasting at least 3 months.

## Introduction

The global lifetime risk of stroke is increasing (1). Despite treatment, stroke patients often remain disabled due to neurological damage. It has been reported that 30–40% of stroke survivors suffer cognitive decline, and these impairments cause disabilities in performing daily living activities and lower quality of life (2). Rehabilitation for stroke patients includes cognitive training based on their implicated domains and severity. Depression after stroke is also known to hinder rehabilitation and worsen outcomes (3). The efficacy of therapeutic interventions for cognition and depression during the acute and subacute phases has been reported (4, 5). However, during the chronic stage, post-stroke cognitive impairment (PSCI) is unlikely to be ameliorated by conventional therapy (6–8). Therefore, new therapeutic measures have been developed for patients with chronic disabilities.

Repetitive transcranial magnetic stimulation (rTMS) is a non-invasive technology that exerts neuromodulating effects and has been applied to treat the cerebral dysfunction caused by various diseases (9, 10). Clinical trials of rTMS for stroke patients have been conducted, reporting therapeutic effects that include recovery from motor weakness, aphasia, and dysphagia (11–13). However, most of these patients were in the acute or subacute phases. Moreover, rTMS has been used to enhance the cognitive function in patients with Alzheimer's dementia and Parkinson's disease (14, 15). However, research on the effect of rTMS treatment on established cognitive impairment after stroke during the chronic phase is insufficient (16).

Above all, rTMS is used worldwide for its apparent effect in major depression. The effect of a protocol applying high-frequency stimulation to the left dorsolateral prefrontal cortex (DLPFC) region is gaining popularity (17), and rTMS' therapeutic effect on post-stroke depression is becoming an established fact. Most of the protocols used involved the administration of a high frequency of 5 Hz or more to the left DLPFC region (18, 19). Nevertheless, there is no definitive protocol for the use of rTMS to enhance cognition in post-stroke patients (20). Our clinical research team established a treatment protocol with high-frequency rTMS over the ipsilesional DLPFC according to our clinical experiences with positive results by retrospective analyses (21). In this study, we aimed to determine whether high-frequency rTMS on the ipsilesional DLPFC had a therapeutic effect in patients with PSCI during the chronic phase of stroke who also exhibited depressive symptoms. As a prospective study, the therapeutic effects were determined *via* psychological and neurobehavioral evaluations.

Despite the widespread application of rTMS in many clinical trials, the mechanisms underlying its therapeutic effects are poorly understood. Recent experimental studies have revealed that rTMS treatment inhibits inflammation and apoptotic cell death while improving the functional recovery in a rat model of focal cerebral ischemia. Among the different mechanisms involved, inflammation is one of the possible targets of rTMS effects, but detailed experimental studies are lacking. To investigate the mechanism of the therapeutic effect of rTMS, the inflammatory status was assessed using peripheral blood samples according to previous findings of augmented inflammatory status in stroke patients (22, 23). Moreover, cognitive functional magnetic resonance imaging (fMRI) findings were obtained from two randomly selected patients to visualize the changes after rTMS treatment in the brain network.

Additionally, to enable comparisons with control stroke patients, a historical control group that had not received rTMS and with available results of follow-up psychological tests was enrolled for retrospective comparative analyses.

## Materials and Methods

### Participants

This prospective clinical trial was approved by the Institutional Review Board (IRB file no: 2018-07-001-015) of the study hospital. The inclusion criteria were as follows: 1) age ≥ 20 years, 2) cognitive impairment developed with stroke despite adequate rehabilitation, and duration of PSCI ≥6 months. In this study, adequate rehabilitation was defined as at least 3 months of intensive rehabilitation consisting of twice of 30 min duration occupational therapy, which essentially includes tailored cognitive and perceptual training. To screen for cognitive impairment, an Mini-Mental State Examination (MMSE) score of 26 (sensitivity, 71%) was selected as the cutoff (24). PSCI was determined based on Diagnostic and Statistical Manual of Mental Disorders, fourth edition (DSM-IV) and Erkinjuntti imaging criteria (see Supplementary Material 1), 3) depression developed after stroke, as determined by a Geriatric Depression Scale (GeDS) score ≥ of 10 or clinical symptoms judged by a medical doctor. Additionally, the complaints of patients and their families regarding disturbances in their quality of life due to PSCI were considered for inclusion. Exclusion criteria were 1) those suspected of other causes of cognitive decline, such as Alzheimer's dementia, 2) those who had previously received rTMS treatment within the preceding 6 months, 3) those suspected of having systemic infections at the time of screening, and 4) contraindications for rTMS (e.g., pacemaker, pregnant, metallic implants such as deep brain stimulation electrode, and cerebral aneurysm clip). Ten patients were recruited from November 2018 to January 2019, and their baseline characteristics are shown in Table 1. Four of these patients had received continuous rehabilitation therapy before the start of treatment, whereas six had not received hospital-based rehabilitation. The treatment settings did not change throughout the study period. All patients were educated on how to perform the cognitive training on their own during the study period.

**Table 1:** Demographic characteristics of the patients.

**Characteristic**	**Values**
**Treatment group (*****N** **=*** **10)**	
Age, years	53.8 ± 8.2 (41–73)*
Gender (Male/Female) (n)	8/2
Education. years	10.4 ± 3.8 (6–16)*
Etiology (n)	10
Cerebral infarction/intracerebral hemorrhage	6/4
**Lesion (n)^a^**	
Right hemisphere	3
- MCA territory, parietal lobe^b^	1
- Basal ganglia and paramedian pons	1
- Internal capsule, posterior limb	1
Left hemisphere	7
- Basal ganglia	2
- Basal ganglia, frontal lobe and thalamus	1
- Basal ganglia, posterior limb of internal capsule and thalamus	1
- Frontal lobe extending to lateral ventricle	1
- ACA territory, corpus callosum genu and parietooccipital lobes	1
- MCA territory, parietofrontotemporal lobes	1
Post-stroke duration, months	29.5 ± 49.5 (6–169)*
Geriatric depression scale (0–30)	16.9 ± 7.1 (7–29)*
Mini-mental state examination (0–30)	22.2 ± 4.7 (12–26)*
Montreal cognitive assessment (0–30)	16.1 ± 5.6 (8–24)*
Intelligence quotient (40–160)	77.6 ± 14.2 (65–100)*
Auditory verbal learning test	60.6 ± 25.3 (31–100)*
Complex figure test	28.7 ± 9.0 (15–41)*
Memory quotient	88.9 ± 19.8 (68–118)*
Global deterioration scale (1–7)	3.9 ± 0.8 (3–5)*
Clinical dementia rating (0–5)	1.0 ± 0.7 (0.5–2)*
Seoul-instrumental activities of daily living (0–45)	25.3 ± 9.1 (15–42)*
Functional ambulation categories (0–5)	3.8 ± 1.6 (1–5)*
**Control group (*****N** **=*** **11)**	
Age, years	61.8 ± 9.7 (52–80)*
Gender (Male/Female) (n) Education. years^c^	8/3 12.2 ± 2.3 (9–16)*
Etiology (n)	11
Cerebral infarction/intracerebral hemorrhage	3/8
**Lesion (n)^a^**	
Right hemisphere	4
- MCA territory, frontoparietoocipital and insula lobe and basal ganglia	1
- Superior frontal lobe	1
- Insula	1
- frontotemporal lobe	1
- Temporal lobe	1
Left hemisphere	6
- External capsule	1
- MCA territory	2
- Basal ganglia	2
- Thalamus	1
Subarachnoid hemorrhage	1
Post-stroke duration, months	18.3 ± 24.6 (6–91)*
Mini-mental state examination (0–30)	21.8 ± 7.7 (4–30)*

Values are given as mean ± SD (range)^*^ except for numbers of patients in etiology and lesion. ACA, anterior cerebral artery; MCA, middle cerebral artery;

a *Brain MRI axial view was attached to the Supplementary Material S5 for each patient*.

b *This patient had infarction, which progressed to multifocal hemorrhagic transformations*.

c *There is no information on the educational level of one patient in the chart review, so the results are for 10 patients*.

### rTMS Procedure

Each patient received a total of 10 days of rTMS, on 5 weekdays per week, for 2 consecutive weeks, using a 70 mm figure-8 coil stimulation device (ALTMS®, Remed Co., Korea). The coil was placed over the ipsilesional DLPFC. The intensity of stimuli was 100% of the patient's resting motor threshold (20 Hz, 5-s train duration, and 55-s intertrain interval) for 20 min (2,000 pulses per session). The resting motor threshold was determined as the minimal intensity required to elicit a potential of > 50 μV peak-to-peak amplitude in the contralateral abductor pollicis brevis muscle at least five out of 10 times. Before the intervention, the resting motor threshold was measured for each patient (see Supplementary Material S2). The protocol was established based on studies of rTMS treatment for depression or cognitive decline, which involved stimulating the DLPFC that transduces an excitatory stimulus with high-frequency (≥5 Hz) stimulation of the cerebral cortex (18, 25). rTMS was administered by trained medical doctors.

### Assessment of Outcomes

The participants underwent baseline psychological tests within 4 weeks before initiation of the rTMS treatment and follow-up tests with the same items 2 and 14 weeks after rTMS initiation. Psychological tests, such as the MMSE (26), Montreal Cognitive Assessment (MoCA) (27), Intelligence Quotient (IQ) from the Wechsler Adult Intelligence Scale Fourth Edition (28), auditory verbal learning test (AVLT), complex figure test (CFT), and memory quotient (MQ), which were derived from the AVLT and CFT performances (29), Global Deterioration Scale (30) and Clinical Dementia Rating – Sum of Boxes (CDR-SB) (31) were used to evaluate cognitive status. The GeDS (32) was also used to assess patients' depressive mood. Two expert clinical psychologists performed cognition and mood evaluations using the authorized nationality-specific versions of the tests.

Several tests for evaluating the motor function, such as the Berg Balance Scale (BBS) (33), trunk impairment scale (34), manual function test (MFT) (35), and Fugl-Meyer Assessment (36), were also conducted by each rehabilitation professional. Additionally, the Stroke Specific Quality of Life Scale (37) was assessed to evaluate satisfaction with the quality of life. The Seoul-Instrumental Activities of Daily Living (38), Modified Barthel Index (39), and Functional Ambulation Categories (40) were used as scales to evaluate daily activities.

Clinical improvement was determined by the significance of the score change as the enrolled patients were diagnosed with chronic PSCI status, where expecting meaningful recovery was difficult (41, 42).

Although the present study was prospectively designed without a control, a retrospective analysis comparing the outcomes in the historical control group was additionally performed using serial MMSE records in the study clinic. All the available data of chronic stroke patients who had received either inpatient or outpatient rehabilitation therapy, from January 2015 to December 2020 with the same criteria as this study enrollment, were collected. A total of 22 patients underwent an MMSE follow-up evaluation with >3 months of interval. Among them, five patients received rTMS treatment around the MMSE evaluation dates, five had participated in another study, and one was in a minimally conscious state. Therefore, these patients were excluded from the additional analyses, and data from 11 patients were used.

### Blood Sampling and Assay for Inflammatory Gene Expression

Morning fasting venous blood for inflammatory cytokine analysis was collected within 4 weeks before (baseline), 2 weeks, and 14 weeks after initiation of the rTMS treatment (immediately after and 12 weeks after completion of the rTMS treatment). The gene expression of inflammatory cytokines, tumor necrosis factor alpha (TNF-α), interleukin (IL)-1β, transforming growth factor beta (TGF-β), and IL-6, was further assessed. Procedures to quantify the mRNA levels of inflammatory cytokines are described in Supplementary Material S3.

Furthermore, to determine whether the patient's basic hematologic status was affected by rTMS treatment, blood samples at baseline and 14 weeks after treatment were compared using the Wilcoxon signed-rank test. Comparisons of nine complete blood count indices (white and red blood cell counts, hemoglobin and hematocrit levels, mean corpuscular volume, mean corpuscular hemoglobin level, red blood cell distribution width, platelet count, and mean platelet volume) and eight chemical analysis indices (sodium, potassium, glucose, chloride, and aspartate transaminase, alanine transaminase, total cholesterol, and triglyceride) were performed (see Supplementary Material S4).

### Acquisition, Processing, and Analysis of fMRI

#### Data Acquisition

Twice fMRI was performed within 4 weeks before the initiation of rTMS and 12 weeks after completion of the treatment. Two randomly selected patients out of the 10 underwent fMRI. For cognition fMRI, the “language sentence completion” task was given, which involves finding appropriate words by showing a sentence with a blank space (for example, “If you go to the mountain, there are ___.” Participants had to generate a word such as “trees” to complete the sentence in mind and not out of mouth). A total of 24 sentences were presented at 5-s intervals, and a 40-s interval was provided for every four sentences.

Scans were obtained using a GE SIGMA 3.0T (General Electric, Milwaukee, Wisconsin). The fMRI sequence parameters were as follows: slice thickness, 4 mm; repetition time [TR], 2,000 ms; echo time [TE], 30 ms; flip angle = 90, matrix = 64 (frequency) × 64 (phase); number of excitations [NEX] = 1, Freq. field of view [FOV] = 24 cm; and phase FOV = 1.0.

#### Data Preprocessing

We corrected the differences in timing across the slices, followed by realigned head movements. We subsequently used images from echo planar imaging for normalization instead of the damaged T1-weighted images (43). The images were smoothed with an isotropic Gaussian kernel (8 mm full width at half maximum). fMRI analysis for significant changes from baseline to 12 weeks after completion of rTMS was conducted using SPM12.

#### General Linear Model

We constructed a general linear model in a task fMRI language sentence completion test. In the patient-level analysis, we used “active condition > rest” as the contrast of interest. Multiple comparison correction was performed using a cluster-extent method with a cluster-forming (uncorrected) *p* threshold of < 0.005.

### Safety Assessment

The patients were monitored from the time of enrollment until completion of the study for any adverse events defined in the Common Terms Criteria for Adverse Events (CTCAE) version 5.0. Reports of adverse events that might be related to the intervention were also available after the study.

### Statistical Analysis

SPSS (IBM, version 21) was used for the data analysis. Using the Wilcoxon signed-rank test, the score from each functional evaluation immediately after rTMS session completion and at 12 weeks after session completion was compared with the baseline score. Statistical significance was set at *p* < 0.05. The changes in MMSE scores of the rTMS treatment group and the historical control group were compared using the Mann-Whitney test.

Changes in cytokine levels after treatment were analyzed using the Wilcoxon signed-rank test, and Spearman correlation analysis was performed to assess the relationship between decrements in the gene expression of each inflammatory cytokine and gains in the score representing cognitive outcome immediately after and after 14 weeks of treatment from the baseline values.

## Results

### Demographic and Clinical Characteristics

The demographic characteristics of the 10 patients (8 men and 2 women) are summarized in Table 1. The mean post-stroke period until enrollment in the study was 29.5 months (ranging from 6 and 169 months), which corresponds to the state where the recovery of function has reached a plateau after stroke. There were 6 cases of cerebral infarction and 4 cases of intracerebral hemorrhage. Lesions were located in the right cerebral hemisphere in three patients and in the left hemisphere in seven. Nine of them received 6 months and one received 3 months of intensive rehabilitation that include cognitive training after acute care of stroke. After the stage, all of them received 10 min duration guidelines for cognitive training at home on every 2–3 months interval outpatient visit. Three patients had diabetes and were taking medication, and one was diagnosed with diabetes at the baseline. Further, three patients with hypertriglyceridemia were reported in the initial evaluation, and all were taking medications to reduce the risk of recurrent stroke before rTMS treatment.

Unfortunately, the clinical psychologist performing the MMSE, MoCA, IQ, MQ, and CDR-SB screening and efficacy evaluations suddenly died due to an emergent disease. For this reason, some data were omitted that include three patients' baseline efficacy evaluations. To compensate for the lost data, the medical records were reviewed, and the evaluation results acquired during routine medical care within the assessment window period were filled with the values. The evaluations were conducted by doctors who passed evaluation training courses, and through this, it was possible to supplement some of the MMSE, GeDS, MoCA, CDR-SB, and Global Deterioration Scale data.

The baseline cognitive score of the patients evaluated through the MMSE was 22.2, and the average ambulatory ability, as seen through the Functional Ambulation Categories score, was 3.8. Among them, 8 patients were evaluated as having the ability to walk on flat ground without aid from other people, with a score of 3 or higher, and the other two patients were evaluated as 1 point.

All patients were diagnosed with depression either by psychological assessments or clinical judgment prior to rTMS intervention, and seven were taking antidepressants. Of the 10 patients with depression, seven were evaluated as mild and three as severe.

In the assessment immediately after completion of the rTMS treatment session, IQ, MQ, AVLT, CFT, SSQoL, and MFT scores were significantly higher than those at baseline (*p* < 0.05). In the assessment conducted 14 weeks after baseline, improvement in cognition seemed obvious, showing sustained increased scores of AVLT, CFT, and MQ, which were increased immediately after completing rTMS treatment (*p* < 0.05). Moreover, MMSE and MoCA scores, which were not increased at 2 weeks after initiation of rTMS, were increased at 14 weeks (*p* < 0.05). CDR-SB showed a significant decrease at the last evaluation (*p* < 0.05). Moreover, MFT scores that were increased immediately after the treatment from baseline also showed a sustained improvement in hand function at the last evaluation along with other motor ability scores, BBS, and trunk impairment scale, which were not increased immediately after treatment (*p* < 0.05; Table 2).

**Table 2:** Changes in scores of functional evaluation after rTMS.

	**Baseline**	**2 weeks**	**14 weeks**	***P*** **-value (** ***n*** **)**	
				**0–2 week**	**0–14 week**
MMSE	23.0 (17–26)	24.7 (18–28)	25.3 (19–30)	0.062 (*7*)	0.041^*^ (7)
MoCA	18.3 (11–24)	19.8 (11–24)	20.3 (11–25)	0.059 (*6*)	0.042^*^ (6)
IQ	79.7 (66–100)	88.2 (72–109)	83.7 (74–99)	0.046^*^ (*6*)	0.058 (*6*)
AVLT	65.5 (34–100)	81.5 (49–117)	87.2 (62–112)	0.042^*^ (*6*)	0.028^*^ (6)
CFT	29.2 (15–41)	35.5 (16–47)	35.2 (18–50)	0.028^*^ (*6*)	0.028^*^ (*6*)
MQ	92.3 (69–118)	104.7 (71–137)	110.7 (87–137)	0.028^*^ (*6*)	0.028^*^ (6)
GlDS	3.5 (3–4)		3.0 (2–4)		0.083 (6)
CDR-SB	5.5 (1–12)		3.75 (0.5–9)		0.018^*^ (*8*)
BBS	47.4 (20–56)	49.4 (22–56)	49.5 (22–56)	0.066 (*10*)	0.042^*^ (*10*)
TIS	16.5 (7–21)	17.1 (8–21)	17.7 (9–23)	0.109 (*10*)	0.026^*^ (*10*)
MFT	63.8 (0–96.88)	67.5 (0–96.88)	67.8 (0–96.88)	0.027^*^ (*10*)	0.027^*^ (*10*)
FMA	48.4 (4–66)	50.4 (4–66)	49.9 (4–66)	0.068 (*10*)	0.066 (*10*)
MBI	74.1 (21–98)	76.5 (33–100)	76.4 (33–100)	0.068 (*10*)	0.168 (*10*)
FAC	3.8 (1–5)	3.9 (1–5)	3.9 (1–5)	0.317 (*10*)	0.317 (*10*)
S-IADL	25.3 (15–42)	23 (15–42)	25 (15–42)	0.285 (*10*)	0.440 (*10*)
SS-QoL	137.1 (61–210)	150.7 (62–245)	149 (62–209)	0.043^*^ (*10*)	0.182 (*10*)
GeDS	18.7 (12–29)	16.7 (3–30)	14.2 (6–29)	0.399 (*6*)	0.136 (6)

*All values are presented a mean (range)*.

* *p < 0.05, Wilcoxon signed-rank test was performed at each follow-up time point compared to the baseline, except for patients who missed even one outcome. GeDS, Geriatric Depression Scale; MMSE, Mini-Mental State Examination; MoCA, Montreal Cognitive Assessment; IQ, Intelligence Quotient; AVLT, auditory verbal learning test; CFT, complex figure test; GlDS, Global Deterioration Scale; CDR-SB, Clinical Dementia Rating – Sum of Boxes; S-IADL, Seoul Instrumental Activities of Daily Living; MMT, Manual Muscle Test; BBS, Berg Balance Scale; TIS, trunk impairment scale; MBI, Modified Barthel Index; SS-QoL, Stroke Specific Quality of Life Scale; MFT, manual function test; FMA, Fugl-Meyer Assessment; FAC, Functional Ambulation Categories. Two weeks indicate immediately after 2-week treatment and 14 weeks indicate 12 weeks after completion of 2-week treatment. (n) means the patient number that was analyzed for change of score from baseline evaluation at each time point*.

While the GeDS scores did not show significant change among all patients, those with moderate-to-severe depression (GeDS ≥ 20) showed amelioration of the score with marginal significance after the treatment (*p* = 0.057). Analyses regarding antidepressant medication status and brain lesion side did not reveal any significance in the affective outcomes of the patients.

A comparison analysis with the historical control group and 11 patients with PSCI (eight men and three women) were conducted. Their mean age was 53.8 ± 8.2 (between 41 and 73), and the mean post-stroke duration was 18.3 ± 24.6 months (between 6 and 91 months) at the time of baseline MMSE assessment (Table 1). The average follow-up evaluation of the control group was 13.7 ± 9.8 months (between 5 and 35 months). The mean baseline MMSE score was 21.8 ± 7.70, which was not different from the patients enrolled in this study. The follow-up score of the historical control group was 22.5 ± 7.57, which did not change during the interval (*p* = 0.356). Although there was no difference in comparison of the changed scores between the control group (0.73 ± 3.90 SD) and the treatment group in this study (2.20 ± 2.04 SD), the patients in the present study showed a significant increment only in the MMSE score 3 months after treatment (*p* < 0.05).

### Changes in fMRI

**Figure 1:**
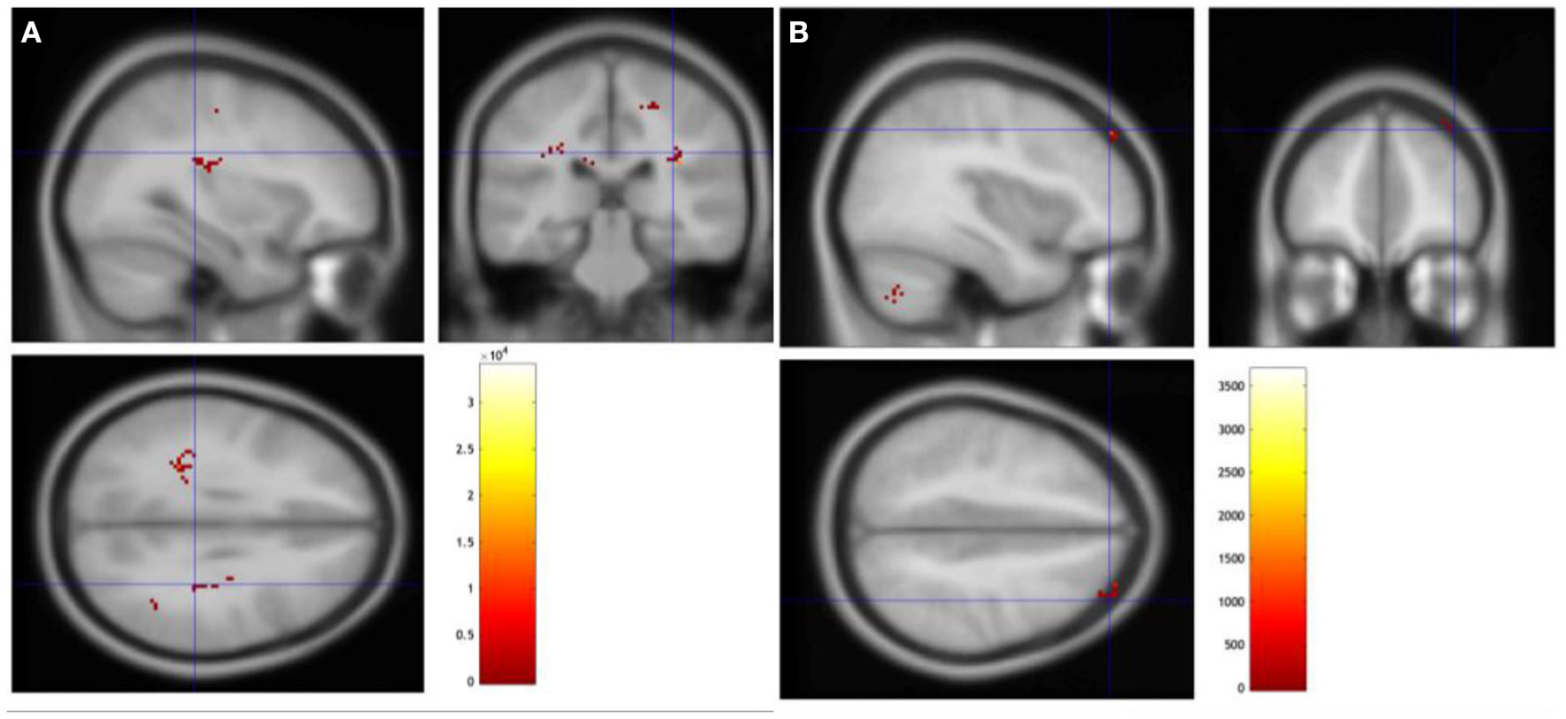
Changes in fMRI during language sentence completion task. **(A)** Change in patient no. 3. **(B)** Change in patient no. 6.

We found brain areas showing greater task-evoked activation after rTMS. More specifically, the right angular gyrus of both participants was activated during the language sentence completion task. Additionally, other brain areas showed greater activation: the right medial frontal gyrus for patient no. 6 (*p* = 0.039); the left supplementary motor area, right hippocampus, right postcentral, left medial frontal, and left postcentral showed greater activation after rTMS for patient no. 3 (*p* = 0.063; Figure 1 and Supplementary Material S6).

### Downregulation of Inflammatory Cytokines and Correlation With Cognitive Improvement

**Figure 2:**
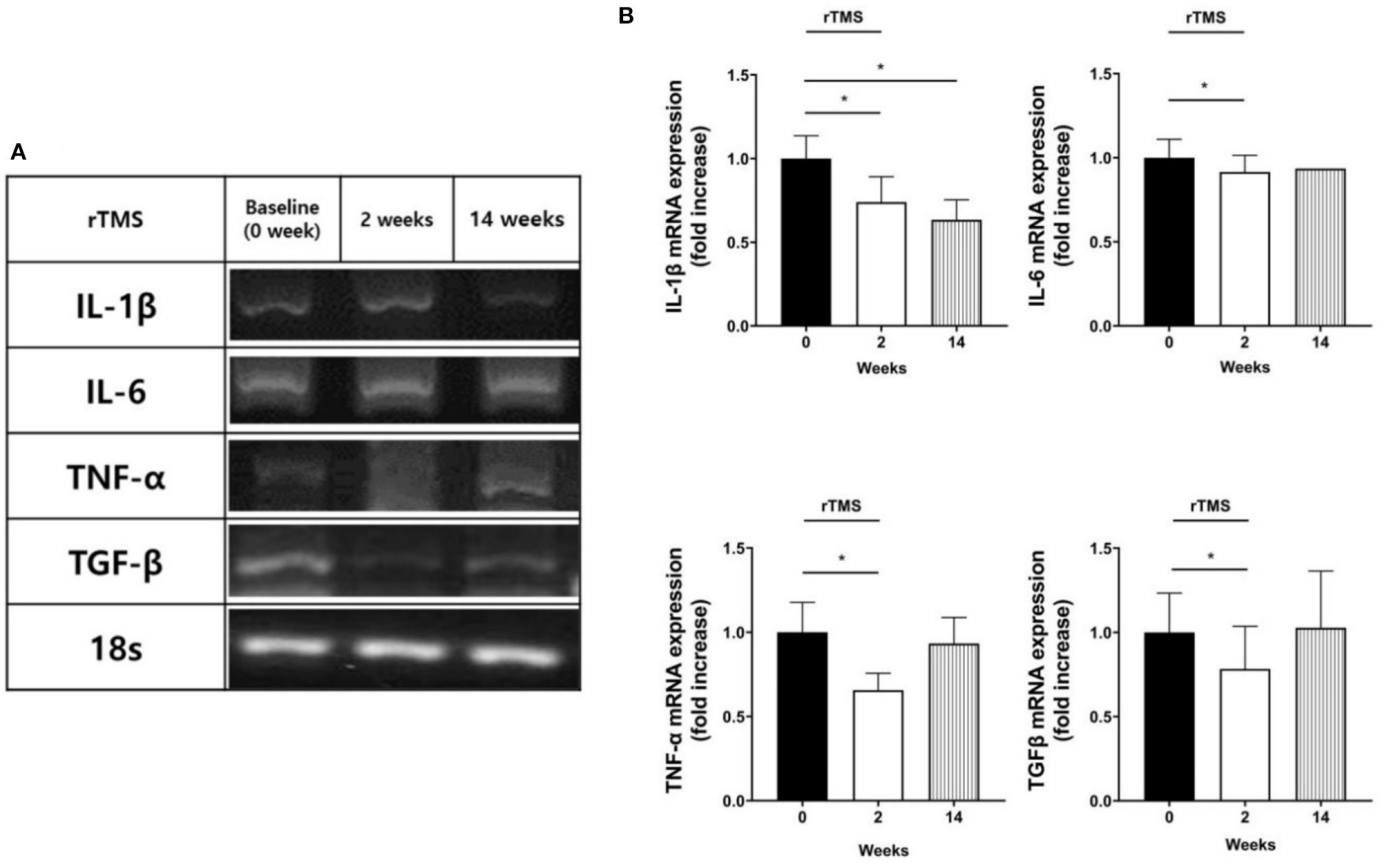
mRNA expression results. Reverse transcription polymerase chain reaction (PCR) products of target mRNAs (IL-1β, IL-6, TNF-α, and TGF-β). **(A)** Representative agarose gel electrophoresis of PCR products on cDNA from human peripheral blood mononuclear cells. **(B)** The relative levels of target mRNA expression. The amount of mRNA expression was quantified by densitometry of bands in comparison to 18s. Densitometry of mRNA band was quantified by three independent scans presented as mean ± standard error (SE) of the mean for ten patients. *p < 0.05; Wilcoxon signed-rank test was used for each follow-up point compared to the baseline.

Reverse transcription polymerase chain reaction (PCR) results from the blood drawn just after the rTMS treatment session indicated downregulated expression of IL-1β, IL-6, TNF-α, and TGF-β mRNA when compared to those before the treatment (*p* < 0.05). Peripheral blood samples drawn 12 weeks after completion of the treatment revealed a sustained reduction in gene expression of IL-1β (*p* < 0.05; Figure 2), while there was no change in C-reactive protein (CRP) level (*p* = 0.838).

**Figure 3:**
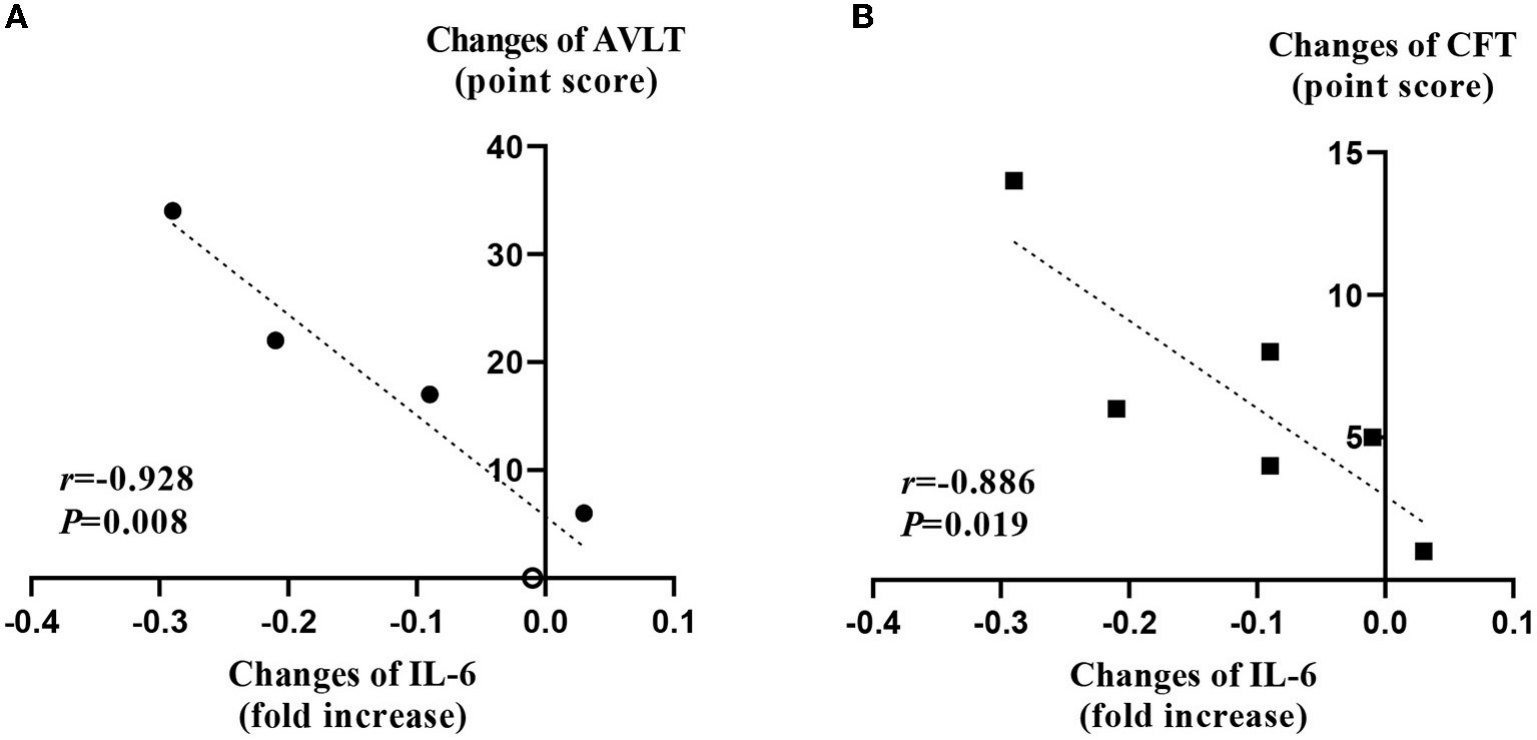
Correlation analysis between changes of interleukin-6 and cognitive function test. **(A)** The change of the IL-6 negatively correlated with the change of the score of AVLT test after 2 weeks of rTMS treatments. The empty circle indicates an overlapped value from two different patients. **(B)** The change of the IL-6 negatively correlated with the change of the score of CFT test after 2 weeks of rTMS treatments. These figures depict results of 6 patients. IL-6, interleukin-6; AVLT, auditory verbal learning test; CFT, complex figure test.

We examined whether the decrease in the expression of each cytokine correlated with changes in cognitive function, depression index, or motor function. The amount of reduction in the mRNA level of IL-6 immediately after the rTMS treatment session from the baseline was highly correlated with increments in AVLT (*r* = 0.928) and CFT (*r* = 0.886), respectively (*p* < 0.05; Figure 3).

### Safety

In the blood test, total cholesterol was slightly higher than the normal range in one patient initially but decreased to the normal range after rTMS treatment. There were no reports of serious adverse events, but some mild (grades 1 and 2) adverse events were observed. Hyperglycemia was reported in two patients without prior diabetes at 14 weeks after initiation of treatment, but they showed improvement at clinical follow-up and did not meet the diagnostic criteria for diabetes. There was an increment of aspartate and alanine aminotransferase in one patient corresponding to CTCAE grade 1 before rTMS treatment, but this was normalized after approximately 1 week of medication.

Moreover, two patients reported constipation symptoms, and one patient each reported vitreal floaters and headaches during the study period. All patients were classified as CTCAE grade 1, which was not directly related to rTMS treatment. Another patient who reported headache had complained of intermittent headache before enrollment in the study. Additionally, one patient reported the appearance of a spider web in her left vision after rTMS. The corresponding ophthalmologist diagnosed a vitreal floater that seemed to have existed before rTMS treatment, without any sign of retinal problems (44, 45), indicating that the patient was able to sense a vitreal floater that was not perceived before treatment.

## Discussion

This study was conducted to determine the possibility of inducing cognitive recovery by using rTMS treatment for patients with PSCI that persisted for more than 6 months, despite intensive rehabilitation treatment for cognition. The results indicated a cognition-enhancing effect in the patients *via* increments in the IQ, AVLT, CFT, and MQ scores just after 2 weeks of rTMS completion. After 3 months, the increments in the AVLT, CFT, and MQ scores were sustained (*p* < 0.05), and the IQ was increased with marginal significance (*p* = 0.058). Moreover, the MMSE and MoCA scores, which did not show changes 2 weeks after the rTMS, were also increased at this time. Furthermore, considering the prevalent knowledge of resistance to treatments in patients with PSCI and that the result also did not show a positive change in the MMSE score in the historical control group, the outcomes in the treatment group seem to be meaningful. According to the changed CDR-SB scores at 3 months after treatment completion, the dementia-ameliorating effect of rTMS seems remarkable. CDR-SB has been acknowledged as a sensitive method for detecting the progression of cognitive impairment (46). The Global Deterioration Scale scores showed only a trend toward ameliorated cognitive dysfunction, and this weak result might have been caused by the sparse rating score system.

With regard to depression, the overall mean scores of the GeDS did not show a significant change in all patients. However, the score decreased in those with severe depression, although with marginal significance. As the efficacy of rTMS treatment has been previously revealed in patients with major depression who do not respond to conventional treatment due to their severe degree of depression, the Food and Drug Administration (FDA) has approved this intervention for such patients (47). Previous studies have also revealed that rTMS monotherapy has a greater antidepressant effect than rTMS add-on therapy (48). Because the total number of participants and non-medicated depressed patients (*n* = 3) was small, the effect on depression could not be fully addressed in this study. Further, the characteristics of patients with substantial cerebral injury could have affected the results.

The BBS, trunk impairment scale, and MFT scores indicating motor function also showed significant improvements after 3 months of treatment completion. Preparation for movement involves the complex and extensive regulation of multiple brain centers (49). The DLPFC plays a crucial role in linking cognition and motor function, and its connections can reportedly reach the primary motor cortex and transfer important information for motor execution (50). Therefore, it can be inferred that the application of rTMS to the DLPFC affected the motor cortex and led to improved motor function.

Regarding the cognition-recovering effect of rTMS in patients with PSCI, the stimulation protocol could be of importance. In the present trial, rTMS was administered to stimulate the DLPFC in the ipsilesional hemisphere at 20 Hz for 10 days. The DLPFC plays an important role in various cognitive processes, such as working memory, planning, banning, and abstract reasoning (11, 51, 52). To date, rTMS studies on cognition have mostly been applied to the left DLPFC, and recent clinical research has reported improvements in the immediate and delayed recall by high-frequency rTMS over the left DLPFC in patients with left hemispheric stroke (53). Although reports on the cognition-enhancing effects of rTMS on the left DLPFC outnumber those of the right side stimulation, as there has been a report of cognitive improvement by rTMS in the right inferior frontal gyrus of patients with Alzheimer's disease or mild cognitive impairment, the application site of rTMS remains controversial (54). Moreover, animal experiments using the ischemic stroke model revealed the efficacy of rTMS at reducing ipsilesional apoptosis, which involves post-stroke neuronal deterioration (55, 56). Along with the abovementioned results, considering that positive effects appeared with increased scores in the cognitive assessments of MMSE, MoCA, IQ, AVLT, CFT, and MQ in this study, high-frequency rTMS on the ipsilesional DLPFC could be a usable protocol for PSCI patients.

Although our sample size was small, the present study provided an understanding of the therapeutic mechanism of rTMS for PSCI. First, an anti-inflammatory effect that spread to the systemic circulation was observed with the decreased gene expression of pro-inflammatory cytokines. Immediately after the rTMS session, the IL-1β, IL-6, TNF-α, and TGF-β levels were decreased, and the depletion of IL-1β was retained 3 months after rTMS. These findings conform with those of previous studies. In a clinical study on elderly patients with refractory depression, serum levels of IL-1β and TNF-α were decreased after rTMS treatment (57). Another study on cerebral infarction also revealed lowered serum levels of IL-6 and TNF-α following rTMS treatment (58). In animal experiments using a brain injury mouse model, rTMS exerted a neurological deficit-ameliorating effect by inhibiting the activity of the TGF-β pathway (59). In the present study, the reduction in the IL-6 gene expression showed a strong correlation with the increments in AVLT (*r* = 0.928) and CFT (*r* = 0.886) scores immediately after the rTMS treatment. This result might be significant in understanding the impact of rTMS on inflammation and the role of rTMS in cognitive impairment in a clinical study that revealed greater cognitive decline at higher IL-6 levels (60). Besides the cytokines we investigated, a previous study showed the possibility of ischemia-induced amyloid beta and tau pathology involvement in the pathogenesis of PSCI (61). Moreover, the impact of inflammatory change in the brain of Alzheimer's dementia has been reported *vice versa* (62). Therefore, investigation of the therapeutic mechanism of rTMS targeting PSCI may adopt biomarkers of Alzheimer's disease in the following research.

Second, according to the fMRI findings, both patients showed increased activity of the right angular gyrus, which is a brain region involved in high-level cognitive functions, such as visuospatial attention, decision-making, solving familiar problems, and reorienting attention to important stimuli (63, 64). Additionally, some activation areas differed between the two patients, which may be due to the differences in the ratio of the functional networks preserved in both hemispheres and the left and right positions of the lesion (65). A previous study reported a decrease in the functional connectivity of the medial prefrontal cortex, left temporal lobe, and hippocampus in patients with PSCI (66, 67). The result suggests that the activation of the right hippocampus and left medial frontal lobe of patient 3 and the right medial frontal area of patient 6 was due to the rTMS treatment. However, this study did not include electroencephalography or other neurophysiological assessment, understanding of baseline mechanism regard to the neural network, such as spike-time dependent plasticity, has not fully been achieved (68). By enlightening the role of neural network in each cognitive function, stimulation of different sites could exert more effect in future research.

In terms of safety, blood chemistry indices reported as abnormal were temporary and could not be regarded as a side effect of rTMS. Instead, the decreasing pattern of total cholesterol was consistent with previous studies showing that rTMS lowers the total cholesterol by altering the lipid metabolism (69). Therefore, chemical confirmation through a large population study is required to confirm these adverse reactions. Consequently, considering the present study along with previous reports, rTMS treatment can be considered a safe treatment technique.

This study had some limitations. First, the sample size was small, with only 10 participants, and the study was conducted without a randomized control group. Furthermore, a learning effect through repeated measurements could not be ruled out. However, other studies have also used MMSE as a short-term cognitive function follow-up assessment tool (70). Repeatable Battery for the Assessment of Neuropsychological Status is known as a tool to exclude content practice effect (71), but since this test is difficult to apply to patients with low cognitive function, other appropriate psychological tests need to be developed and used for short-term evaluation studies. Nevertheless, differential results at 2 and 14 weeks after the initiation of rTMS, such as the significantly increased MMSE and MoCA scores at the last examination and not immediately after the treatment, suggest a real improvement in cognition.

Studies with larger sample sizes with matched control groups and using cognitive evaluation tools to avoid learning effects should be conducted.

## Conclusions

In summary, we have obtained significant results that suggest that high-frequency rTMS treatment for ipsilateral DLPFC may exert beneficial effects on the short- and long-term improvement of cognitive function in chronic PSCI patients by reducing inflammation in the brain and altering the functional connectivity of several brain regions.

## Data Availability Statement

The raw data supporting the conclusions of this article will be made available by the authors, without undue reservation.

## Ethics Statement

The studies involving human participants were reviewed and approved by IRB file No: 2018-07-001-015. The patients/participants provided their written informed consent to participate in this study.

## Author Contributions

MK designed the study and performed critical revision of the article. BC conducted clinical research and drafted the manuscript. JK and JMK involved in the statistical analysis. J-WC and JCho have quantified pro-inflammatory cytokines using reverse transcription polymerase chain reaction. KK and JCha analyzed fMRI and describe the corresponding results. All authors contributed to the article and approved the submitted version.

## Funding

This research was supported by a grant from the Korea Health Technology R&D Project through the Korea Health Industry Development Institute (KHIDI), funded by the Ministry of Health & Welfare, Republic of Korea (Grant Number: HI16C1559).

## Conflict of Interest

The authors declare that the research was conducted in the absence of any commercial or financial relationships that could be construed as a potential conflict of interest.

## Publisher's Note

All claims expressed in this article are solely those of the authors and do not necessarily represent those of their affiliated organizations, or those of the publisher, the editors and the reviewers. Any product that may be evaluated in this article, or claim that may be made by its manufacturer, is not guaranteed or endorsed by the publisher.

## References

[1] Gorelick PB. The global burden of stroke: persistent and disabling. Lancet Neurol. (2019) 18:417–8. 10.1016/S1474-4422(19)30030-4 30871943

[2] Leys D Hénon H Mackowiak-Cordoliani M-A Pasquier F. Poststroke dementia. Lancet Neurol. (2005) 4:752–9. 10.1016/S1474-4422(05)70221-0 16239182

[3] Pohjasvaara T Vataja R Leppävuori A Kaste M Erkinjuntti T. Depression is an independent predictor of poor long-term functional outcome post-stroke. Eur J Neurol. (2001) 8:315–9. 10.1046/j.1468-1331.2001.00182.x 11422427

[4] Rabadi MH Rabadi FM Edelstein L Peterson M. Cognitively impaired stroke patients do benefit from admission to an acute rehabilitation unit. Arch Phys Med Rehabil. (2008) 89:441–8. 10.1016/j.apmr.2007.11.014 18295621

[5] Wang S-B Wang Y-Y Zhang Q-E Wu S-L Ng CH Ungvari GS. Cognitive behavioral therapy for post-stroke depression: a meta-analysis. J Affect Disord. (2018) 235:589–96. 10.1016/j.jad.2018.04.011 29704854

[6] Cicerone KD Langenbahn DM Braden C Malec JF Kalmar K Fraas M. Evidence-based cognitive rehabilitation: updated review of the literature from 2003 through 2008. Arch Phys Med Rehabil. (2011) 92:519–30. 10.1016/j.apmr.2010.11.015 21440699

[7] Paolucci S. Role, indications, and controversies of antidepressant therapy in chronic stroke patients. Eur J Phys Rehabil Med. (2013) 49:233. 23558703

[8] Paolucci S Antonucci G Gialloreti E Traballesi M Lubich S Pratesi L. Predicting stroke inpatient rehabilitation outcome: the prominent role of neuropsychological disorders. Eur Neurol. (1996) 36:385–90. 10.1159/000117298 8954308

[9] Lefaucheur J-P André-Obadia N Antal A Ayache SS Baeken C Benninger DH. Evidence-based guidelines on the therapeutic use of repetitive transcranial magnetic stimulation (rTMS). Clin Neurophysiol. (2014) 125:2150–206. 10.1016/j.clinph.2014.05.021 25034472

[10] Lefaucheur J-P Aleman A Baeken C Benninger DH Brunelin J Di Lazzaro V. Evidence-based guidelines on the therapeutic use of repetitive transcranial magnetic stimulation (rTMS): an update (2014–2018). Clin Neurophysiol. (2020) 131:474–528. 10.1016/j.clinph.2020.02.003 31901449

[11] Barbey A Barsalou L. Reasoning and problem solving: models. Encycl Neurosci. (2009) 8:35–43. 10.1016/B978-008045046-9.00435-6

[12] Du J Yang F Liu L Hu J Cai B Liu W. Repetitive transcranial magnetic stimulation for rehabilitation of poststroke dysphagia: a randomized, double-blind clinical trial. Clin Neurophysiol. (2016) 127:1907–13. 10.1016/j.clinph.2015.11.045 26778719

[13] Zhang L Xing G Fan Y Guo Z Chen H Mu Q. Short-and long-term effects of repetitive transcranial magnetic stimulation on upper limb motor function after stroke: a systematic review and meta-analysis. Clin Rehabil. (2017) 31:1137–53. 10.1177/0269215517692386 28786336

[14] Ahmed MA Darwish ES Khedr EM Ali AM. Effects of low versus high frequencies of repetitive transcranial magnetic stimulation on cognitive function and cortical excitability in Alzheimer's dementia. J Neurol. (2012) 259:83–92. 10.1007/s00415-011-6128-4 21671144

[15] Jiang Y Guo Z McClure MA He L Mu Q. Effect of rTMS on Parkinson's cognitive function: a systematic review and meta-analysis. BMC Neurol. (2020) 20:1–14. 10.1186/s12883-020-01953-4 33076870 PMC7574251

[16] Liu M Bao G Bai L Yu E. The role of repetitive transcranial magnetic stimulation in the treatment of cognitive impairment in stroke patients: a systematic review and meta-analysis. Sci Prog. (2021) 104:00368504211004266. 10.1177/00368504211004266 33827345 PMC10455033

[17] Berlim MT McGirr A Beaulieu M-M Turecki G. High frequency repetitive transcranial magnetic stimulation as an augmenting strategy in severe treatment-resistant major depression: a prospective 4-week naturalistic trial. J Affect Diso. (2011) 130:312–7. 10.1016/j.jad.2010.10.011 21056475

[18] Gu SY Chang MC. The effects of 10-Hz repetitive transcranial magnetic stimulation on depression in chronic stroke patients. Brain Stimul. (2017) 10:270–4. 10.1016/j.brs.2016.10.010 27839722

[19] Frey J Najib U Lilly C Adcock A. Novel TMS for stroke and depression (NoTSAD): accelerated repetitive transcranial magnetic stimulation as a safe and effective treatment for post-stroke depression. Front Neurol. (2020) 11:788. 10.3389/fneur.2020.00788 32849235 PMC7431489

[20] Kim BR Kim D-Y Chun MH Yi JH Kwon JS. Effect of repetitive transcranial magnetic stimulation on cognition and mood in stroke patients: a double-blind, sham-controlled trial. Am J Phys Med Rehabilit. (2010) 89:362–8. 10.1097/PHM.0b013e3181d8a5b1 20407301

[21] Kim M Kim JM Kim J Cha B. Effect of cognition by repetitive transcranial magnetic stimulation on ipsilesional dorsolateral prefrontal cortex in subacute stroke patients. Front Neurol. (2022) 54. 10.3389/fneur.2022.823108 35185773 PMC8848770

[22] Huang J Upadhyay UM Tamargo RJ. Inflammation in stroke and focal cerebral ischemia. Surg Neurol. (2006) 66:232–45. 10.1016/j.surneu.2005.12.028 16935624

[23] Sasso V Bisicchia E Latini L Ghiglieri V Cacace F Carola V. Repetitive transcranial magnetic stimulation reduces remote apoptotic cell death and inflammation after focal brain injury. J Neuroinflammation. (2016) 13:1–6. 10.1186/s12974-016-0616-5 27301743 PMC4908713

[24] Dong Y Slavin MJ Chan BP-L Venketasubramanian N Sharma VK Collinson SL. Improving screening for vascular cognitive impairment at three to six months after mild ischemic stroke and transient ischemic attack. Int Psychoger. (2014) 26:787. 10.1017/S1041610213002457 24423626

[25] Speer AM Repella JD Figueras S Demian NK Kimbrell TA Wasserman EM. Lack of adverse cognitive effects of 1 Hz and 20 Hz repetitive transcranial magnetic stimulation at 100% of motor threshold over left prefrontal cortex in depression. J ECT. (2001) 17:259–63. 10.1097/00124509-200112000-00005 11731727

[26] Kang Y Na DL Hahn S. A validity study on the Korean Mini-Mental State Examination (K-MMSE) in dementia patients. J Korean Neurol Assoc. (1997) 15:300–8.16778398

[27] Lee J-Y Lee DW Cho S-J Na DL Jeon HJ Kim S-K. Brief screening for mild cognitive impairment in elderly outpatient clinic: validation of the Korean version of the Montreal Cognitive Assessment. J Geriatr Psychiatry Neurol. (2008) 21:104–10. 10.1177/0891988708316855 18474719

[28] Hwang S Kim J Park K Chey J Hong S. Korean Wechsler Adult Intelligence Scale-IV. Daegu: Korea Psychology. (2012).

[29] Kim H. Rey-Kim Memory Test: Manual. Neuropsychology Publishing Co, Daegu, Korea p. (1999).

[30] Choi SH Na DL Lee BH Hahm DS Jeong JH Jeong Y. The validity of the Korean version of global deterioration scale. J Korean Neurol Assoc. (2002) 20:612–7.30906387

[31] Choi SH Na DL Lee BH Hahm DS Jeong JH Yoon SJ. Estimating the validity of the Korean version of expanded clinical dementia rating (CDR) scale. J Korean Neurol Assoc. (2001) 19:585–91.

[32] Kim JY Park JH Lee JJ Huh Y Lee SB Han SK. Standardization of the Korean version of the geriatric depression scale: reliability, validity, and factor structure. Psychiat Investig. (2008) 5:232. 10.4306/pi.2008.5.4.232 20046343 PMC2796007

[33] Jung HY Park JH Shim JJ Kim MJ Hwang MR Kim SH. Reliability test of Korean version of berg balance scale. J Korean Acad Rehabilit Med. (2006) 30:611–8.

[34] Seo H-D Kim N-J Chung Y-J. Reliability of the Korean version of the trunk impairment scale in patients with stroke. Phys Ther Korea. (2008) 15:87–96.

[35] Chai K Lee H. Assessment of upper extremity function in normal korean adults by manual function test. J Kor Soc Occup Ther. (1997) 5:52–7.

[36] Kim H Her J Ko J Park D-s Woo J-H You Y. Reliability, concurrent validity, and responsiveness of the Fugl-Meyer Assessment (FMA) for hemiplegic patients. J Phys Ther Sci. (2012) 24:893–9. 10.1589/jpts.24.893

[37] Kim M Lee Y. Reliability and validity of the Korean version of stroke-specific quality of life questionnaire. J Neurosci Nurs. (2021) 53:49–54. 10.1097/JNN.0000000000000560 33156133

[38] Ku HM Kim JH Kwon EJ Kim SH Lee HS Ko HJ. A study on the reliability and validity of Seoul-Instrumental Activities of Daily Living (S-IADL). J Korean Neuropsych Assoc. (2004) 43:189–99.

[39] Jung HY Park BK Shin HS Kang YK Pyun SB Paik NJ. Development of the Korean version of Modified Barthel Index (K-MBI): multi-center study for subjects with stroke. J Korean Acad Rehabilit Med. (2007) 31:283–97.

[40] Collen FM Wade DT Bradshaw CM. Mobility after stroke: reliability of measures of impairment and disability. Int Disabil Stud. (1990) 12:6–9. 10.3109/03790799009166594 2211468

[41] Zheng F Yan L Zhong B Yang Z Xie W. Progression of cognitive decline before and after incident stroke. Neurology. (2019) 93:e20–e8. 10.1212/WNL.0000000000007716 31127071

[42] Levine DA Galecki AT Langa KM Unverzagt FW Kabeto MU Giordani B. Trajectory of cognitive decline after incident stroke. JAMA. (2015) 314:41–51. 10.1001/jama.2015.6968 26151265 PMC4655087

[43] Calhoun VD Wager TD Krishnan A Rosch KS Seymour KE Nebel MB. The impact of T1 versus EPI spatial normalization templates for fMRI data analyses. Wiley Online Library. (2017) 38:5331–42. 10.1002/hbm.23737 28745021 PMC5565844

[44] Kung S Ahuja Y Iezzi R Sampson SM. Posterior vitreous detachment and retinal tear after repetitive transcranial magnetic stimulation. Brain Stimul. (2011) 4:218–21. 10.1016/j.brs.2011.08.007 21930451

[45] Marafon SB Miron J-P Juncal VR Figueiredo N Downar J Blumberger DM. Retinal tear and posterior vitreous detachment following repetitive transcranial magnetic stimulation for major depression: a case report. Brain Stimul. (2020) 13:467–9. 10.1016/j.brs.2019.12.017 31884185

[46] Jeong JH Na HR Choi SH Kim J Na DL Seo SW. Group-and home-based cognitive intervention for patients with mild cognitive impairment: a randomized controlled trial. Psychother Psychosom. (2016) 85:198–207. 10.1159/000442261 27230861

[47] Jorge RE Robinson RG Tateno A Narushima K Acion L Moser D. Repetitive transcranial magnetic stimulation as treatment of poststroke depression: a preliminary study. Biol Psychiat. (2004) 55:398–405. 10.1016/j.biopsych.2003.08.017 14960293

[48] Slotema CW Dirk Blom J Hoek HW Sommer IE. Should we expand the toolbox of psychiatric treatment methods to include Repetitive Transcranial Magnetic Stimulation (rTMS)? A meta-analysis of the efficacy of rTMS in psychiatric disorders. J Clin Psychiat. (2010) 71:873. 10.4088/JCP.08m04872gre 20361902

[49] Cohen O Sherman E Zinger N Perlmutter S Prut Y. Getting ready to move: transmitted information in the corticospinal pathway during preparation for movement. Curr Opin Neurobiol. (2010) 20:696–703. 10.1016/j.conb.2010.09.001 20926287 PMC3153449

[50] Cao N Pi Y Liu K Meng H Wang Y Zhang J. Inhibitory and facilitatory connections from dorsolateral prefrontal to primary motor cortex in healthy humans at rest—An rTMS study. Neurosci Lett. (2018) 687:82–7. 10.1016/j.neulet.2018.09.032 30243883

[51] Curtis CE D'Esposito M. Persistent activity in the prefrontal cortex during working memory. Trends Cogn Sci. (2003) 7:415–23. 10.1016/S1364-6613(03)00197-9 12963473

[52] Goel V Dolan RJ. Differential involvement of left prefrontal cortexin inductive and deductive reasoning. Cognition. (2004) 93:B109–B21. 10.1016/j.cognition.2004.03.001 15178381

[53] Tsai P-Y Lin W-S Tsai K-T Kuo C-Y Lin P-H. High-frequency versus theta burst transcranial magnetic stimulation for the treatment of poststroke cognitive impairment in humans. J Psychiat Neurosci. (2020) 45:190060. 10.1503/jpn.190060 32159313 PMC7828923

[54] Eliasova I Anderkova L Marecek R Rektorova I. Non-invasive brain stimulation of the right inferior frontal gyrus may improve attention in early Alzheimer's disease: a pilot study. J Neurol Sci. (2014) 346:318–22. 10.1016/j.jns.2014.08.036 25216556

[55] Guo F Lou J Han X Deng Y Huang X. Repetitive transcranial magnetic stimulation ameliorates cognitive impairment by enhancing neurogenesis and suppressing apoptosis in the hippocampus in rats with ischemic stroke. Front Physiol. (2017) 8:559. 10.3389/fphys.2017.00559 28824455 PMC5539749

[56] Hong J Chen J Li C An D Tang Z Wen H. High-frequency rTMS improves cognitive function by regulating synaptic plasticity in cerebral ischemic rats. Neurochem Res. (2021) 46:276–86. 10.1007/s11064-020-03161-5 33136229

[57] Zhao X Li Y Tian Q Zhu B Zhao Z. Repetitive transcranial magnetic stimulation increases serum brain-derived neurotrophic factor and decreases interleukin-1β and tumor necrosis factor-α in elderly patients with refractory depression. J Int Med Res. (2019) 47:1848–55. 10.1177/0300060518817417 30616482 PMC6567781

[58] Ge L Zhao Y-X Chang Y-X Cui W-S Zhai X-Z Ma Q-Y. Effect of low-rTMS in combined with edaravone on the inflammatory cytokines and cerebral metabolites in patients with cerebral infarction and aphasia. J Hainan Med Univ. (2017) 23:132–5.

[59] Manaenko A Lekic T Barnhart M Hartman R Zhang JH. Inhibition of transforming growth factor-β attenuates brain injury and neurological deficits in a rat model of germinal matrix hemorrhage. Stroke. (2014) 45:828–34. 10.1161/STROKEAHA.113.003754 24425124 PMC3966308

[60] Economos A Wright CB Moon YP Rundek T Rabbani L Paik MC. Interleukin 6 plasma concentration associates with cognitive decline: the northern Manhattan study. Neuroepidemiology. (2013) 40:253–9. 10.1159/000343276 23364322 PMC3725587

[61] Chi N-F Chao S-P Huang L-K Chan L Chen Y-R Chiou H-Y. Plasma amyloid beta and tau levels are predictors of post-stroke cognitive impairment: a longitudinal study. Front Neurol. (2019) 10:715. 10.3389/fneur.2019.00715 31312178 PMC6614443

[62] Rui W Xiao H Fan Y Ma Z Xiao M Li S. Systemic inflammasome activation and pyroptosis associate with the progression of amnestic mild cognitive impairment and Alzheimer's disease. J Neuroinflammation. (2021) 18:1–14. 10.1186/s12974-021-02329-2 34856990 PMC8638109

[63] Clemens B Jung S Mingoia G Weyer D Domahs F Willmes K. Influence of anodal transcranial direct current stimulation (tDCS) over the right angular gyrus on brain activity during rest. PLoS ONE. (2014) 9:e95984. 10.1371/journal.pone.0095984 24760013 PMC3997501

[64] Studer B Cen D Walsh V. The angular gyrus and visuospatial attention in decision-making under risk. Neuroimage. (2014) 103:75–80. 10.1016/j.neuroimage.2014.09.003 25219333

[65] Bartolomeo P de Schotten MT. Let thy left brain know what thy right brain doeth: Inter-hemispheric compensation of functional deficits after brain damage. Neuropsychologia. (2016) 93:407–12. 10.1016/j.neuropsychologia.2016.06.016 27312744

[66] Andrews-Hanna JR Reidler JS Sepulcre J Poulin R Buckner RL. Functional-anatomic fractionation of the brain's default network. Neuron. (2010) 65:550–62. 10.1016/j.neuron.2010.02.005 20188659 PMC2848443

[67] Tuladhar AM Snaphaan L Shumskaya E Rijpkema M Fernandez G Norris DG. Default mode network connectivity in stroke patients. PLoS ONE. (2013) 8:e66556. 10.1371/journal.pone.0066556 23824302 PMC3688936

[68] Markram H Gerstner W. Sjo stro m PJ. Spike-timing-dependent plasticity: a comprehensive overview. Front Synapt Neurosci. (2012) 4. 10.3389/fnsyn.2012.00002 22807913 PMC3395004

[69] Ren W Ma J Li J Zhang Z Wang M. Repetitive transcranial magnetic stimulation (rTMS) modulates lipid metabolism in aging adults. Front Aging Neurosci. (2017) 9:334. 10.3389/fnagi.2017.00334 29089885 PMC5650987

[70] Jia Y Xu L Yang K Zhang Y Lv X Zhu Z. Precision repetitive transcranial magnetic stimulation over the left parietal cortex improves memory in Alzheimer's disease: a randomized, double-blind, sham-controlled study. Front Aging Neurosci. (2021) 348. 10.3389/fnagi.2021.693611 34267648 PMC8276073

[71] Randolph C Tierney MC Mohr E Chase TN. The Repeatable Battery for the Assessment of Neuropsychological Status (RBANS): preliminary clinical validity. J Clin Exp Neuropsychol. (1998) 20:310–9. 10.1076/jcen.20.3.310.823 9845158

