# Supplementary material for: Therapeutic Effect of Repetitive Transcranial Magnetic Stimulation for Post-stroke Vascular Cognitive Impairment: A Prospective Pilot Study
Source: Front Neurol. 2022 Mar 22;13:813597. doi: 10.3389/fneur.2022.813597 (PMC8980431; doi:10.3389/fneur.2022.813597)
Supplement: Supplementary file 1 [file Table_1.DOCX]

**[ Supplementary Material ]**

**Modified NINDS-AIREN criteria for subcortical ischemic vascular dementia**

1. predominantly white matter cases characterized by extending periventricular and deep white matter lesions: extending caps or irregular halo (>10 mm broad) and diffuse confluent hyperintensities (>25 mm) and at least 1 lacunar infarct in the deep gray matter
2. predominant “lacunar cases” in which multiple lacunes ≥ 5 in the deep gray matter were associated with at least moderate white matter lesions.

Erkinjuntti T et al. Research criteria for subcortical vascular dementia in clinical trials. *J Neural Transm Suppl.* 2000;59:23–30.
