# Supplementary material for: Therapeutic Effect of Repetitive Transcranial Magnetic Stimulation for Post-stroke Vascular Cognitive Impairment: A Prospective Pilot Study
Source: Front Neurol. 2022 Mar 22;13:813597. doi: 10.3389/fneur.2022.813597 (PMC8980431; doi:10.3389/fneur.2022.813597)
Supplement: Supplementary file 2 [file Table_2.DOCX]

**[ Supplementary Material ] The patients’ resting motor threshold**

| **Subject** | **Stimulation side** | **Baseline** | **End of treatment** | ***P*** |
| --- | --- | --- | --- | --- |
| 1 | Right | 25* | 25* |  |
| 2 | Left | 34* | 34* |  |
| 3 | Right | 32* | 32* |  |
| 4 | Right | 30* | 30* |  |
| 5 | Left | 32* | 30* |  |
| 6 | Left | 32* | 32* |  |
| 7 | Left | 30* | 32* |  |
| 8 | Left | 32* | 32* |  |
| 9 | Left | 36* | 34* |  |
| 10 | Left | 36* | 36* |  |
| Total |  | 31.9±3.2** | 31.7±3.0** | 0.564† |
| Motor-evoked potentials was recorded from abductor pollicis brevis muscle.  * % of maximum output of the stimulator  ** Mean±SD  † Wilcoxon signed rank test was performed for investigate the differences before and after treatment. | | | | |
