# Supplementary material for: Therapeutic Effect of Repetitive Transcranial Magnetic Stimulation for Post-stroke Vascular Cognitive Impairment: A Prospective Pilot Study
Source: Front Neurol. 2022 Mar 22;13:813597. doi: 10.3389/fneur.2022.813597 (PMC8980431; doi:10.3389/fneur.2022.813597)
Supplement: Supplementary file 3 [file Table_3.DOCX]

**[ Supplementary Material ]** **Procedures to quantify mRNA levels of inflammatory cytokines**

Peripheral blood mononuclear cells, which are mostly lymphocytes and monocytes, were separated from the whole blood by a density gradient centrifugation method using Ficoll-paque plus (GE healthcare, USA). Total RNA was extracted from the peripheral blood mononuclear cells using TRIZOL reagent according to the manufacture’s protocol (Thermo Fisher Scientific, USA) (72). After extraction with chloroform, the RNA was precipitated from the aqueous phase by adding isopropanol and washed with 75% ethanol. Final RNA was dissolved in DEPC-treated water and stored at -75℃. Reverse transcription of the total RNA was used to synthesize the first strand cDNA using Maxime RT PreMix (Oligo dT primer) (iNtRON bio, South Korea). The final reaction concentration was prepared as follows: a reaction mixture containing Accupower Taq PCR Master Mix (Bioneer, South Korea), 100 ng of cDNA sample, Diethyl pyrocarbonate water and 10 pmol of each primer for the appropriate target sequence in 20 uL total reaction volume. The gene-specific primers used are listed in below in a Table. Each PCR amplification cycling (denaturation, annealing, extension) conditions were as follows: 95℃ for 1 min, 95℃ for 30 sec, 62℃ 30 sec, 72℃ 30 sec, 35 cycle for TNF-α, IL-1β, and IL-6 and 95℃ for 1 min, and 95℃ for 30 sec, 58℃ 30 sec, 72℃ 30 sec, 35 cycle for TGF- β and 18s using Veriti 96 well thermal cycler (Applied biosystems, USA). PCR products (5-10 uL) were separated by electrophoresis through 1.5% agarose gel (Biosciences, USA) at 80 V for 20 min in Tris acetate/EDTA buffer with gelRed Loading view (BioMax, South Korea). The intensity of each PCR product was evaluated semiquantitatively using a UV transilluminator light box and densitometric scanning analyzing software (TL120 software, Nonlinear Dynamics, Newcastle, UK).

**The gene-specific primers for reverse transcription PCR**

| Gene | Prime sequences (5’-3’) | Product length (bp) |
| --- | --- | --- |
| TNF-α | Sense: GAGTGACAAGCCTGTAGCCCATGTTGTAGCA Antisense: GCAATGATCCCAAAGTAGACCTGCCCAGACT | 444 |
| IL-1β | Sense: AAACAGATGAAGTGCTCCTTCCAGG Antisense: TGGAGAACACCAACTTGTTGCTCCA | 391 |
| TGB-β | Sense: CTCGCCAGAGTGGTTATCTT Antisense: AGTGTGTTATCCCTGCTGTCA | 123 |
| IL-6 | Sense: ATGAACTCCTTCTCCACAAGCGC Antisense: GAAGAGCCCTCAGGCTGGACTG | 628 |
| 18s | Sense: CCGCAGCTAGGAATAATGGA Antisense: CCCTCTTAATCATGGCCTCA | 76 |
