# Supplementary material for: Therapeutic Effect of Repetitive Transcranial Magnetic Stimulation for Post-stroke Vascular Cognitive Impairment: A Prospective Pilot Study
Source: Front Neurol. 2022 Mar 22;13:813597. doi: 10.3389/fneur.2022.813597 (PMC8980431; doi:10.3389/fneur.2022.813597)
Supplement: Supplementary file 4 [file Table_4.DOCX]

**[ Supplementary Material ]** **Result of hematology and blood chemistry**

|  | **Units** | **Baseline** | **End of treatment** | ***p*** |
| --- | --- | --- | --- | --- |
| Hematology | | | | |
| White blood cells (WBC) | x 10^3^ cells/㎕ | 6.35±1.48 | 6.71±2.06 | 0.386 |
| Red blood cells (RBC) | x 10^6^ cells/㎕ | 4.56±0.38 | 4.57±0.51 | 0.878 |
| Hemoglobin (Hgb) | g/dL | 14.1±1.06 | 14.1±1.55 | 0.767 |
| Hematocrit (Hct) | % | 41.5±3.03 | 42.1±4.36 | 0.440 |
| Mean corpuscular volume (MCV) | fL | 91.2±2.45 | 92.2±2.57 | 0.092 |
| Mean corpuscular hemoglobin (MCH) | Pg | 31.1±0.82 | 30.9±1.02 | 0.138 |
| Red blood cell distribution width (RDW) | % | 12.8±0.37 | 12.7±0.62 | 0.725 |
| Platelet count (PLT) | x 10^3^ cells/㎕ | 253.5±69.9 | 258.0±77.2 | 0.760 |
| Mean platelet volume (MPV) | fL | 9.88±0.60 | 9.68±0.70 | 0.095 |
| Blood chemistry | | | | |
| Sodium (Na) | mEq/L | 141.1±2.51 | 139.5±1.65 | 0.011* |
| Potassium (K) | mEq/L | 4.35±0.30 | 4.43±0.40 | 0.250 |
| Chloride (Cl) | mEq/L | 103.5±2.17 | 101.4±1.78 | 0.011* |
| Glucose | mg/dL | 131.8±44.3 | 146.5±82.2 | 0.575 |
| Aspartate transaminase (AST) | IU/L | 23.7±10.7 | 20.4±5.23 | 0.414 |
| Alanine transaminase (ALT) | IU/L | 23.9±13.2 | 20.6±6.02 | 0.953 |
| Total cholesterol | mg/dL | 152.3±32.1 | 137.7±20.8 | 0.017* |
| Triglyceride | mg/dL | 158.8±59.5 | 170.7±94.6 | 0.878 |

All values are presented a mean±SD.

*p<0.05, Wilcoxon signed rank test was performed.
