# Supplementary material for: Therapeutic Effect of Repetitive Transcranial Magnetic Stimulation for Post-stroke Vascular Cognitive Impairment: A Prospective Pilot Study
Source: Front Neurol. 2022 Mar 22;13:813597. doi: 10.3389/fneur.2022.813597 (PMC8980431; doi:10.3389/fneur.2022.813597)
Supplement: Supplementary file 5 [file Table_5.DOCX]

**[Supplementary Material 5]** **Brain MRI axial view for each patient**

| Treatment group | | | | |
| --- | --- | --- | --- | --- |
|  | Patient No.1 | Patient No.2 | Patient No.3 | Patient No.4 |
| MRI | 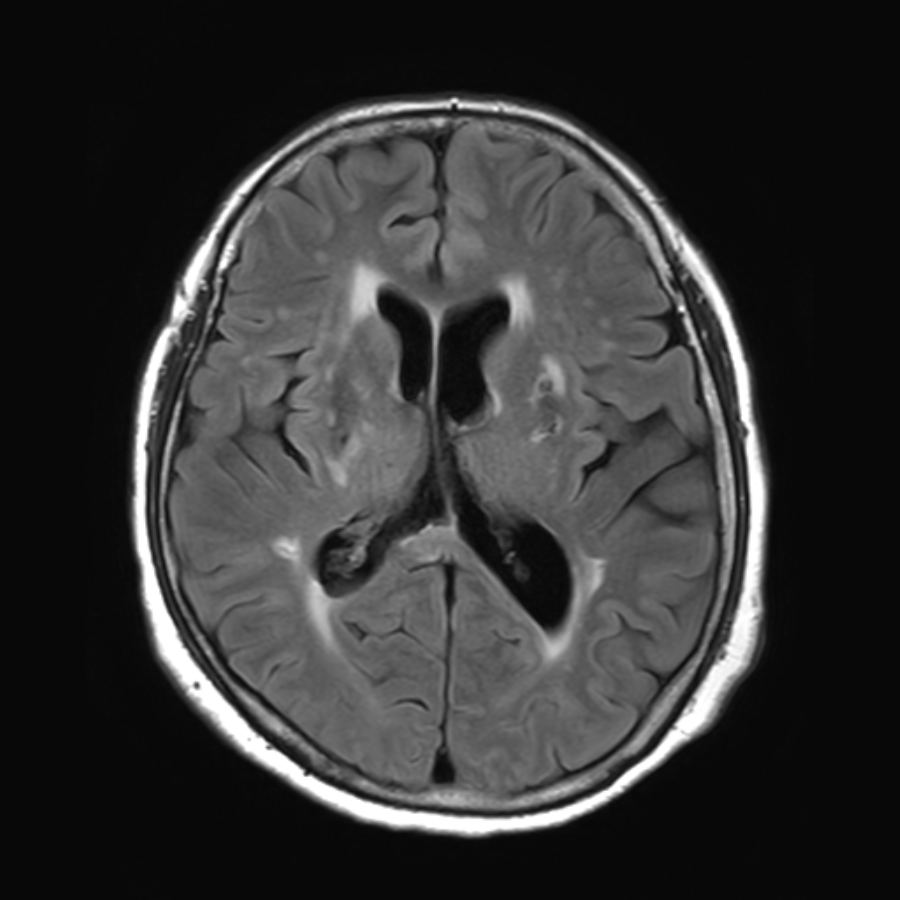 | 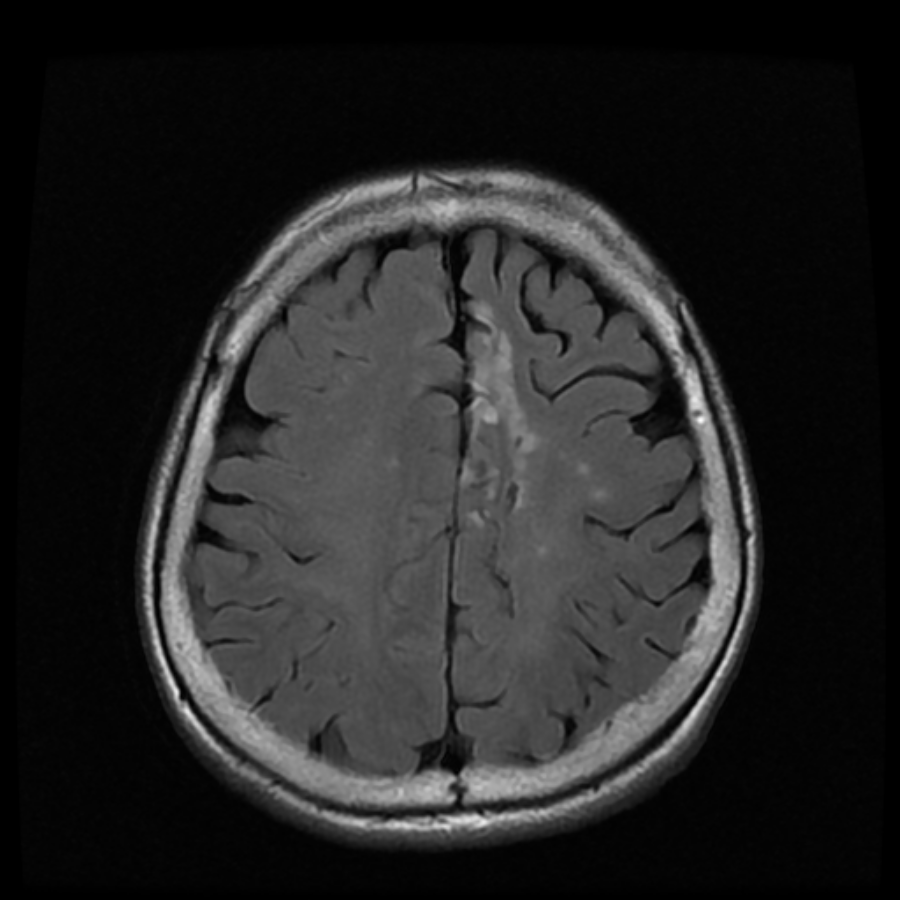 | 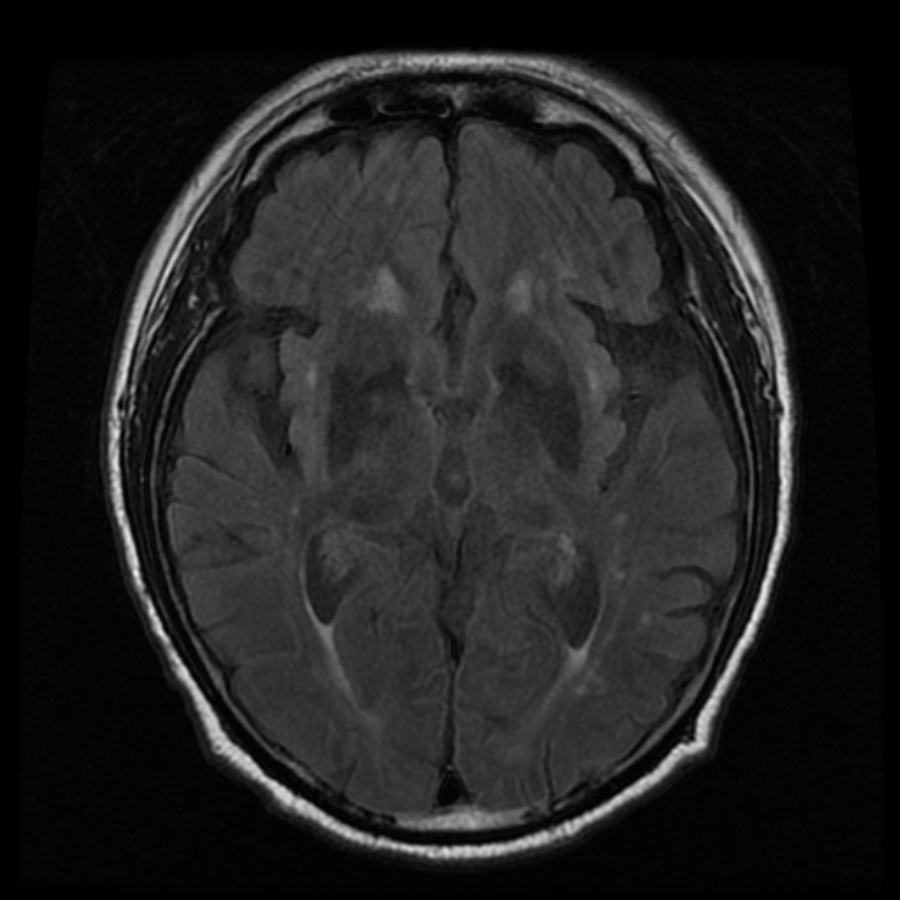 | 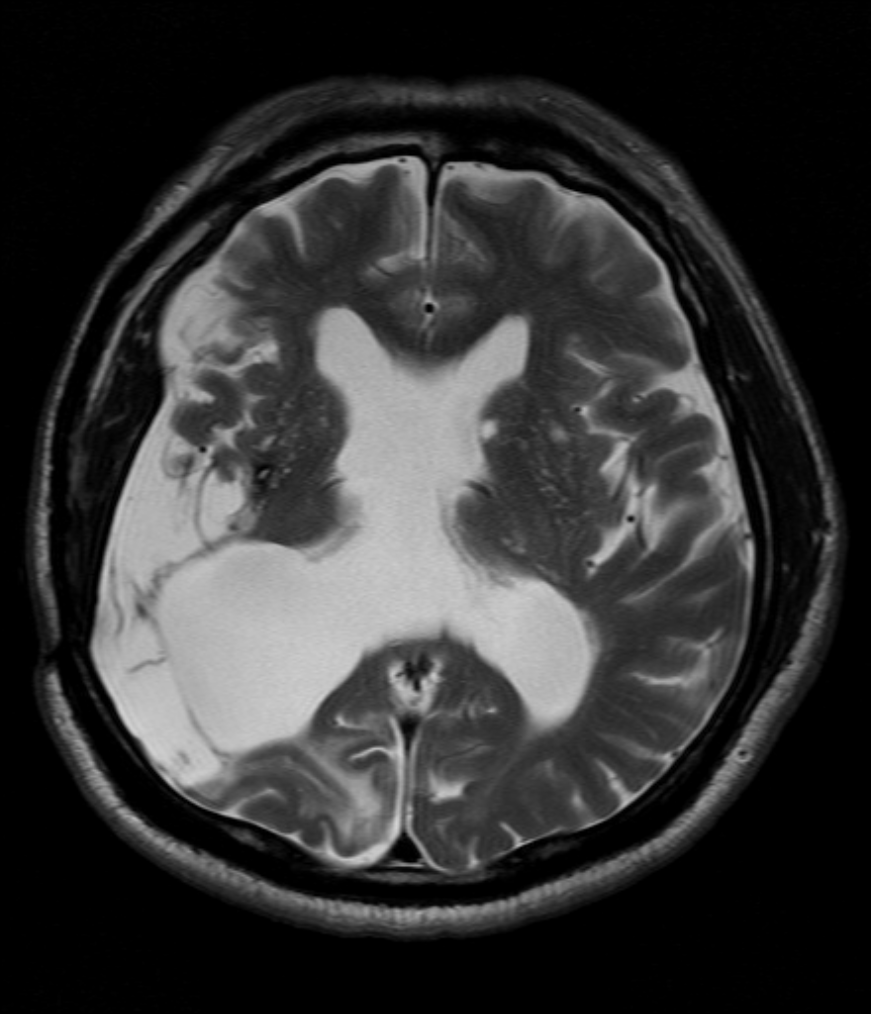 |
| Lesion | Rt.basal ganglia infarction | Lt.ACA territory and left parietooccipital lobes infarction | Rt.basal ganglia and paramedian pons infarction | Rt. MCA territory infarction with multifocal hemorrhagic transformations |
|  | Patient No.5 | Patient No.6 | Patient No.7 | Patient No.8 |
| MRI | 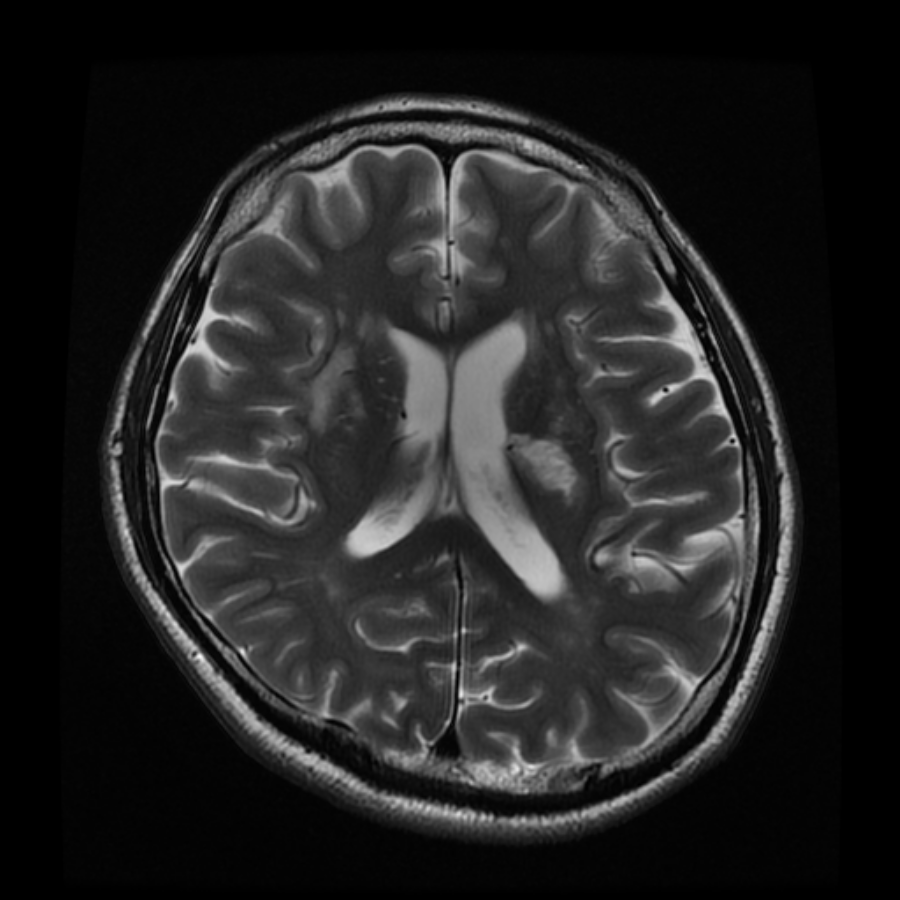 | 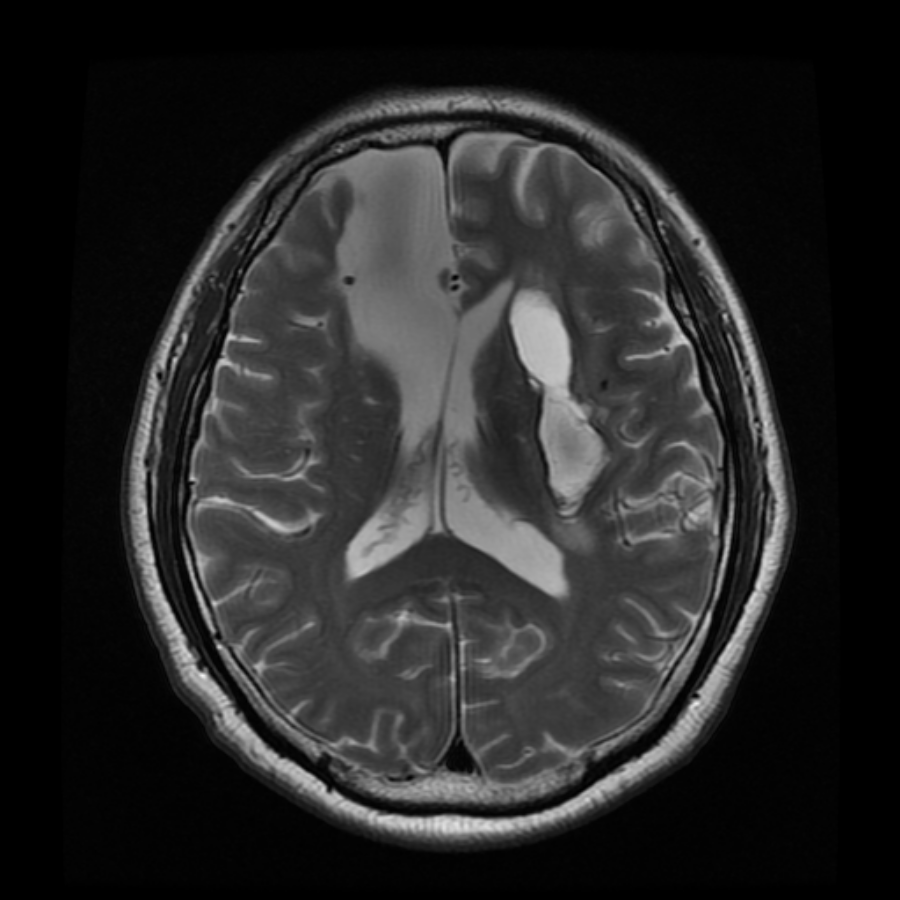 | 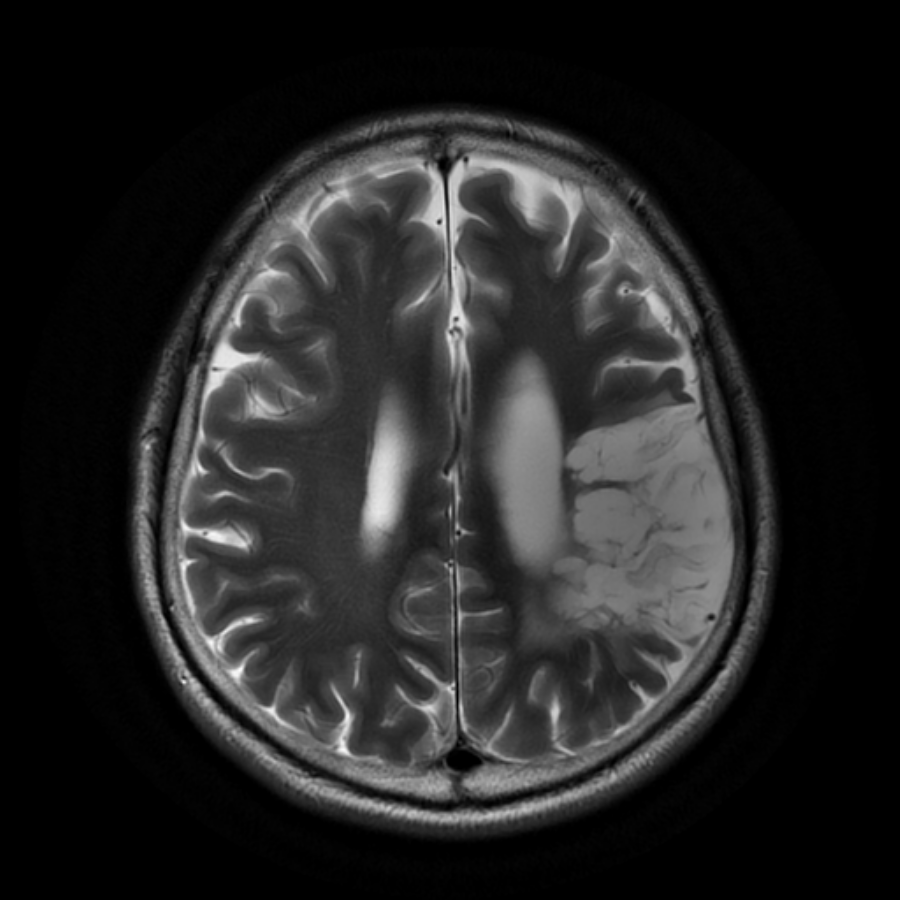 | 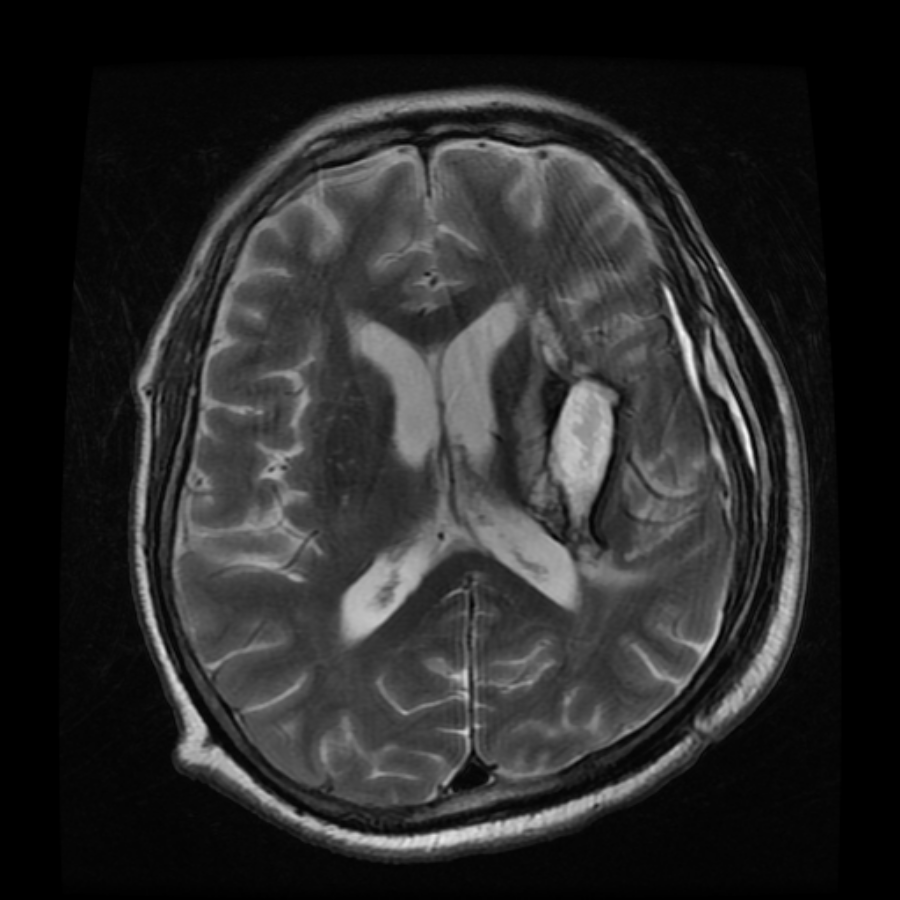 |
| Lesion | Lt.basal ganglia infarction | Lt.basal ganglia  hemorrhage with Rt.prefrontal lobe  porecephalic cyst formation | Lt.frontoparietotemporal lobes infarction with ipsilateral Wallerian degeneration | Lt.basal ganglia - frontal lobe - thalamus hemorrhage |
|  | Patient No.9 | Patient No.10 |  | |
| MRI | 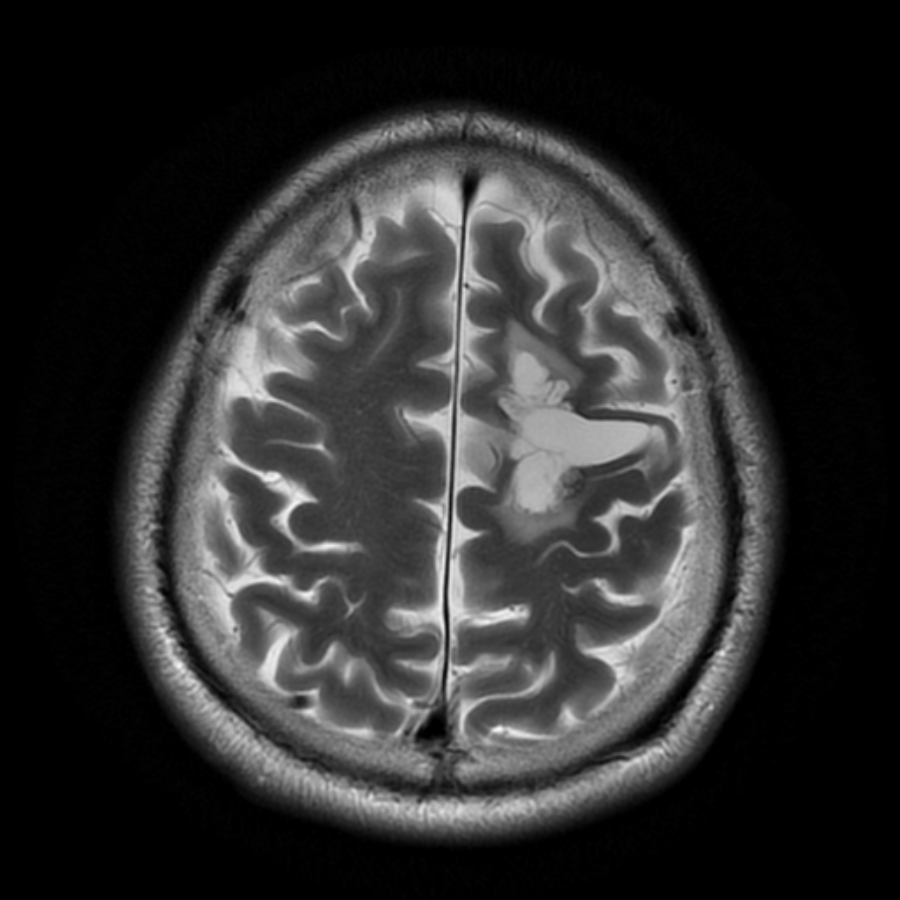 | 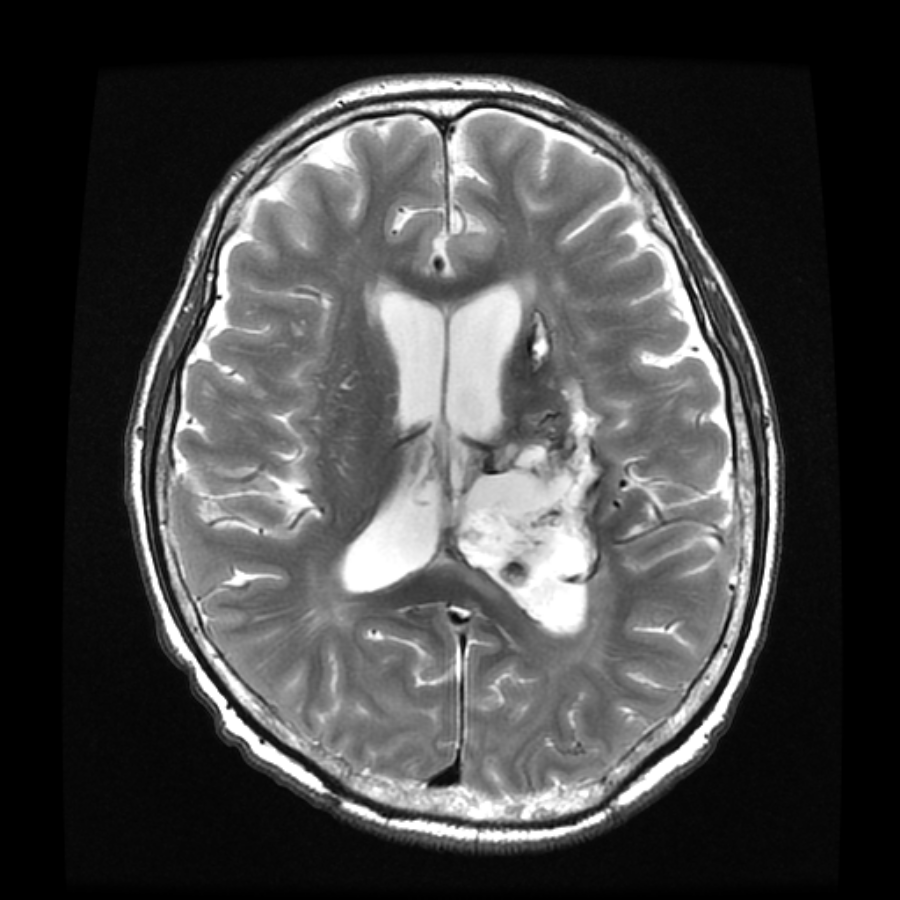 |  | |
| Lesion | Lt.frontal lobe extending to lateral ventricle hemorrhage with encephalomalacia | Lt.posterior basal ganglia –posterior limb of internal capsule posterior portion - thalamus, and another one in Lt.anterior basal ganglia hemorrhage |  |  |

| Control group | | | | |
| --- | --- | --- | --- | --- |
|  | Patient 1 | Patient 2 | Patient 3 | Patient 4 |
| MRI | 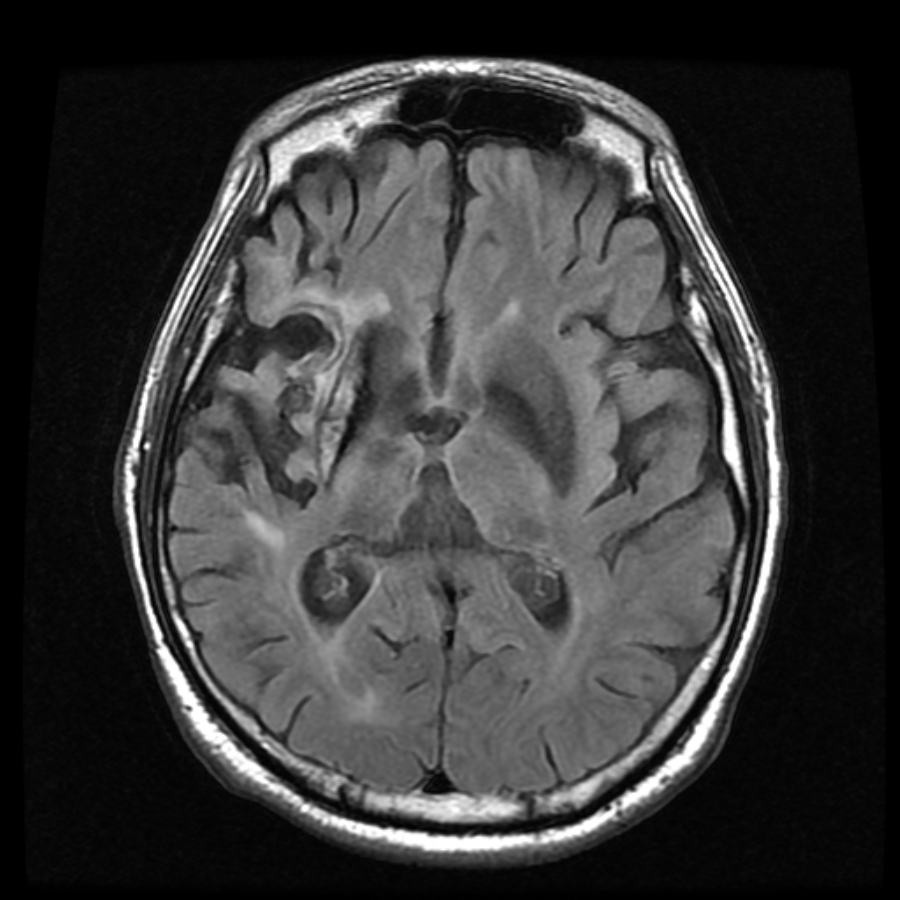 | 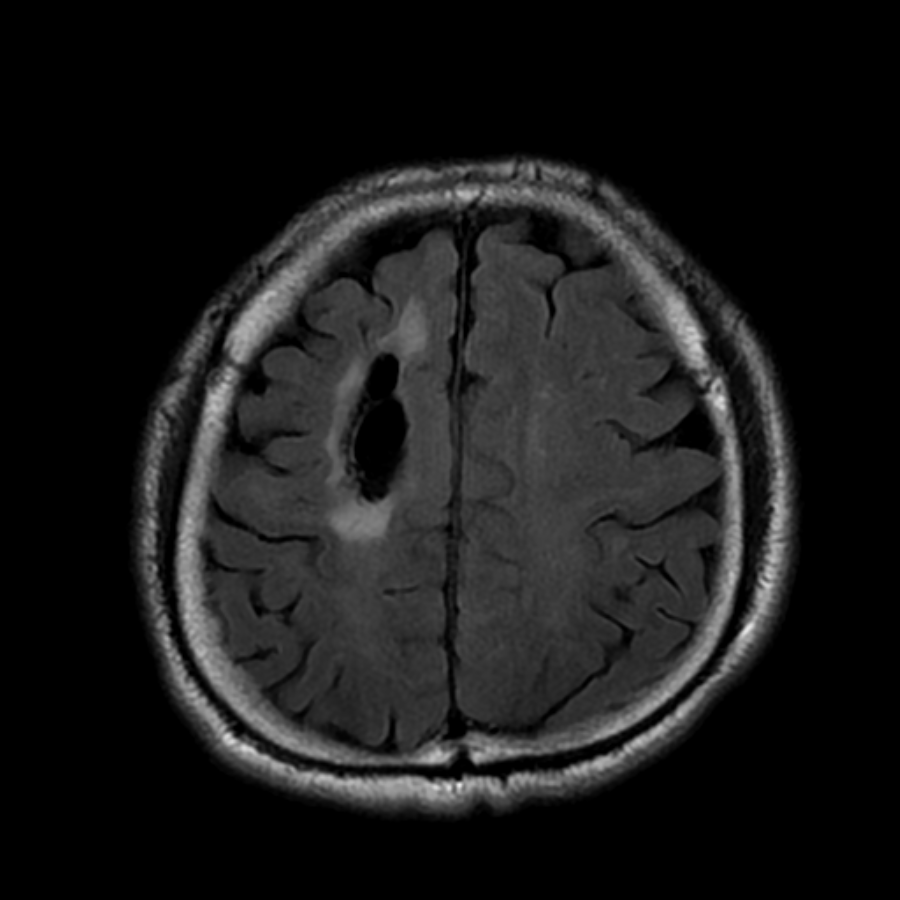 | 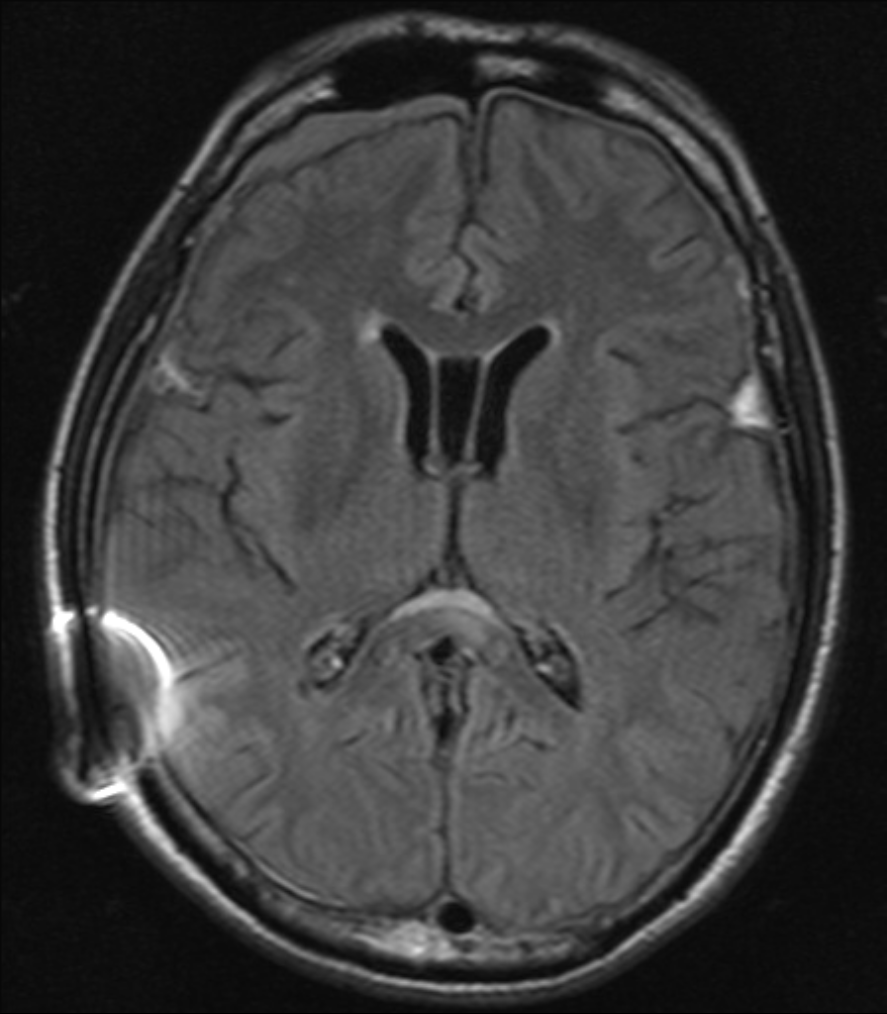 | 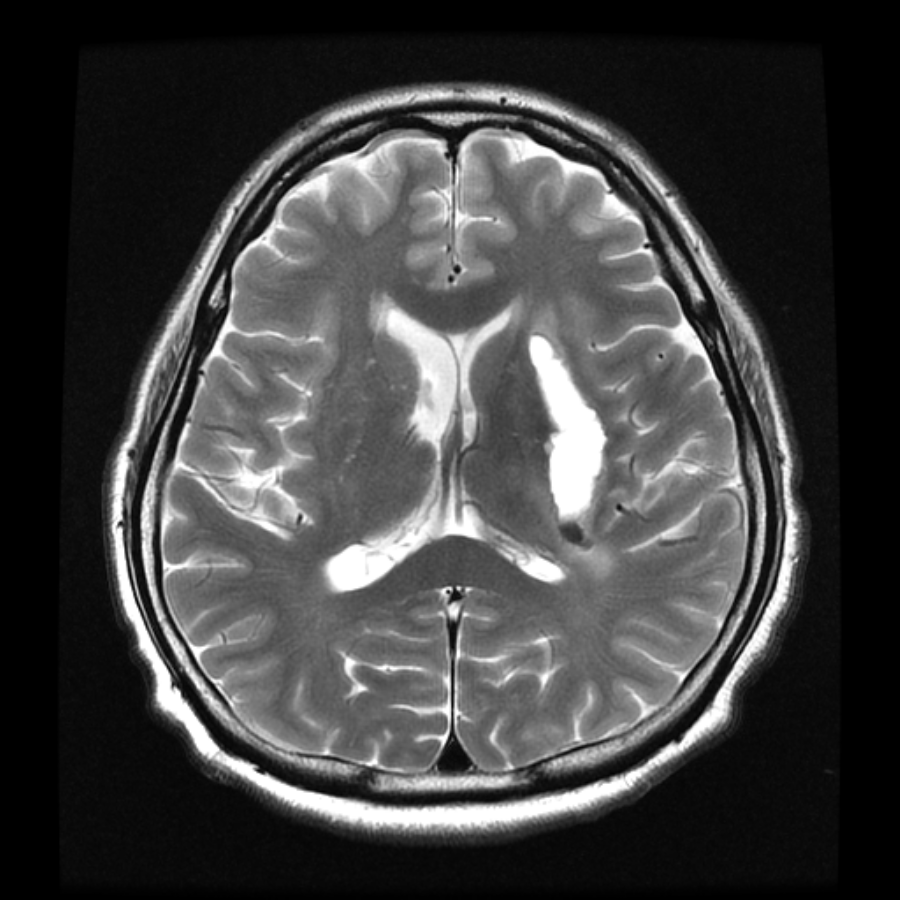 |
| Lesion | Rt. MCA territory infarction, frontoparietotemporal and insula lobe and basal ganglia | Rt. intracerebral hemorrhage in superior frontal white matter, intraventricular hemorrhage | Subarachnoid hemorrhage and focal encephalomalacia at splenium of corpus callosum. | Lt. external capsule hemorrhage |
|  | Patient 5 | Patient 6 | Patient 7 | Patient 8 |
| MRI | 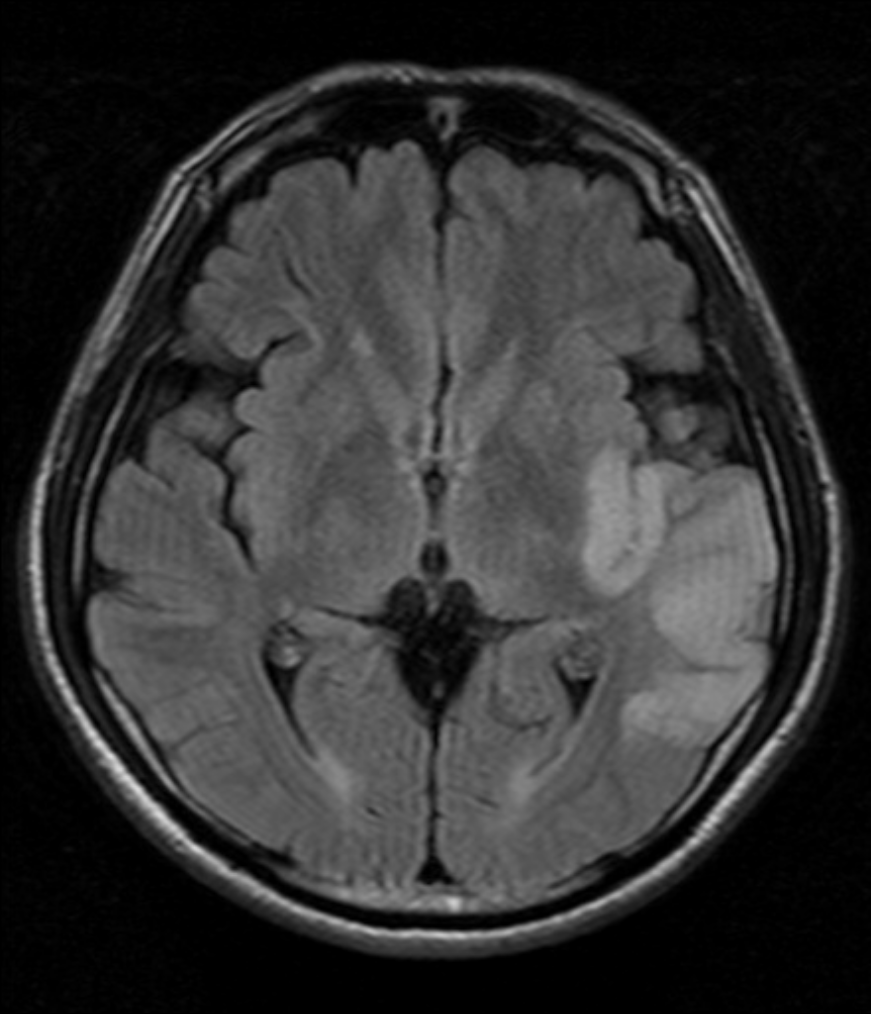 | 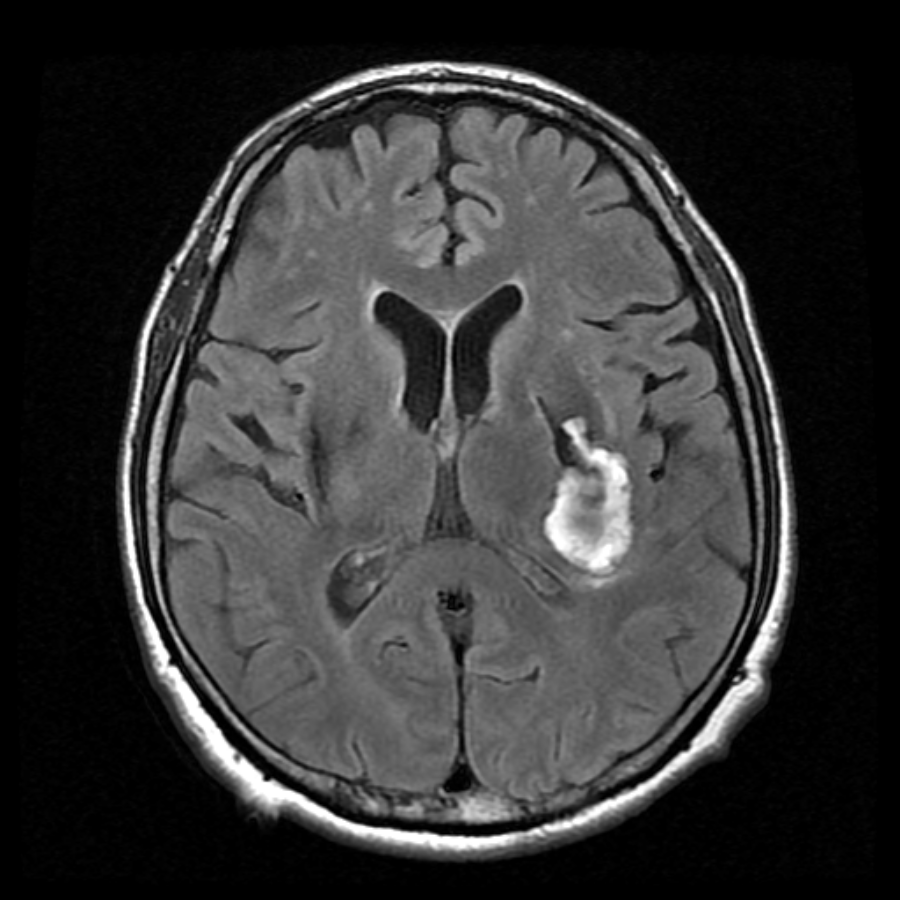 | 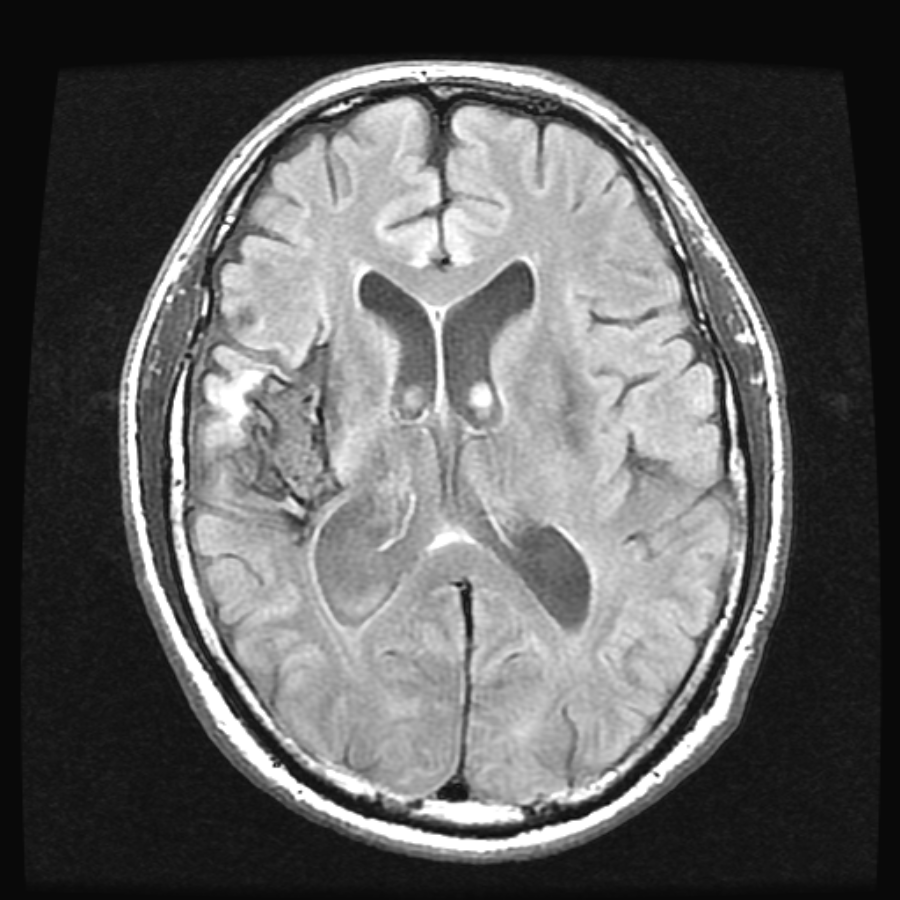 | 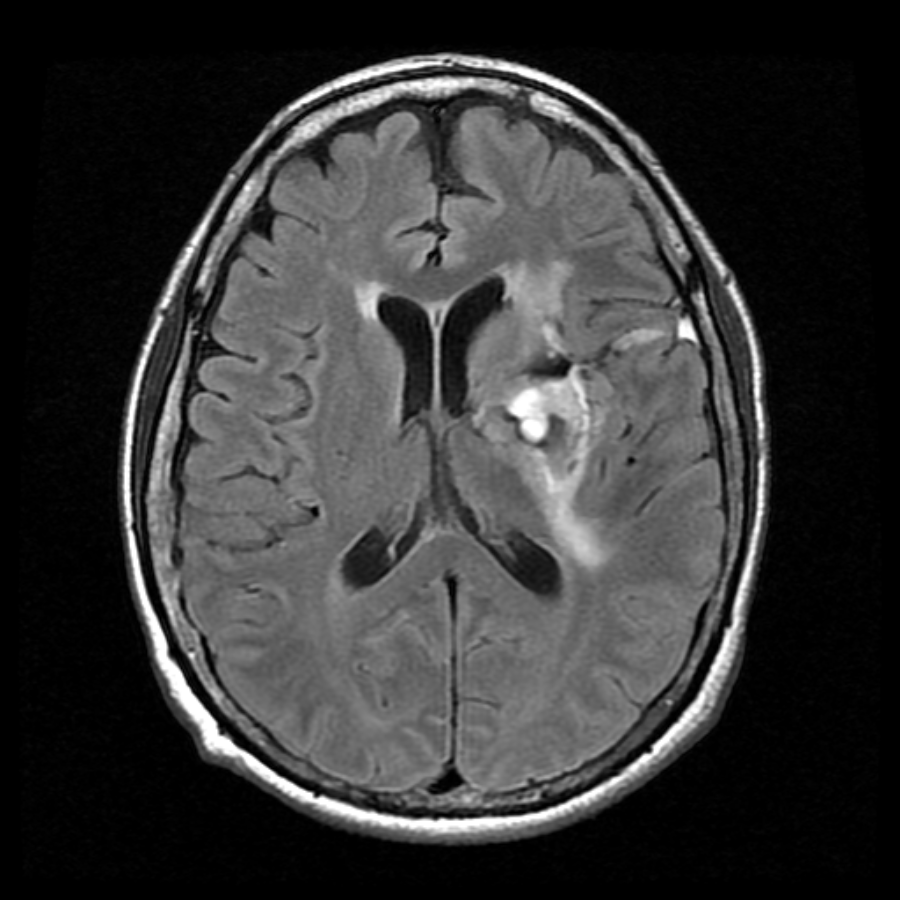 |
| Lesion | Lt. MCA infarction, parietotemporal branch | Lt. basal ganglia hemorrhage | Rt. insula - frontotemporal lobe hemorrhage with encephalomalatic cavity | Lt. basal ganglia hemorrhage with diffuse subarachnoid hemorrhage |
|  | Patient 9 | Patient 10 | Patient 11 |  |
| MRI | 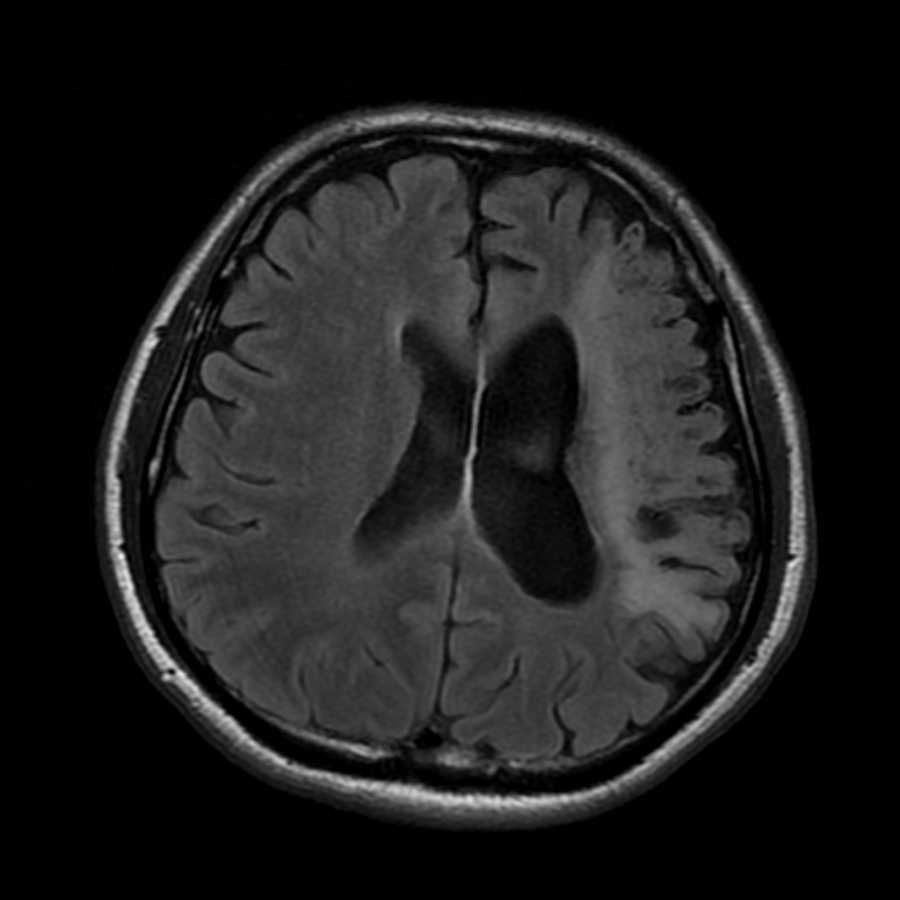 | 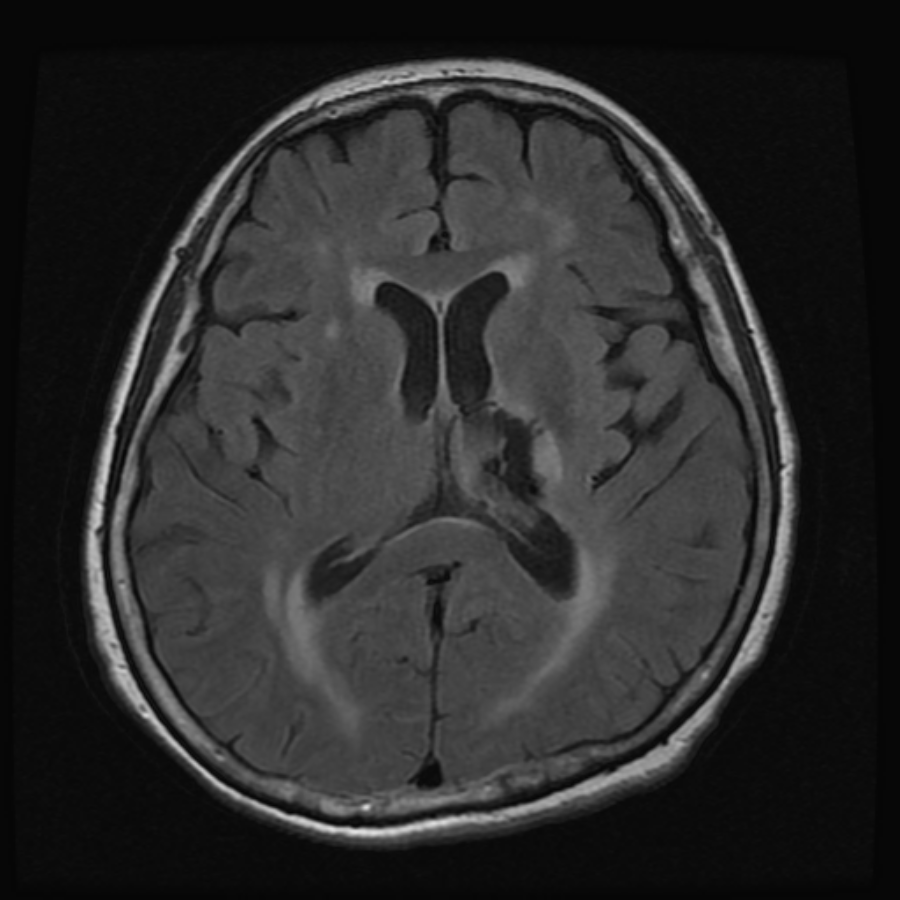 | 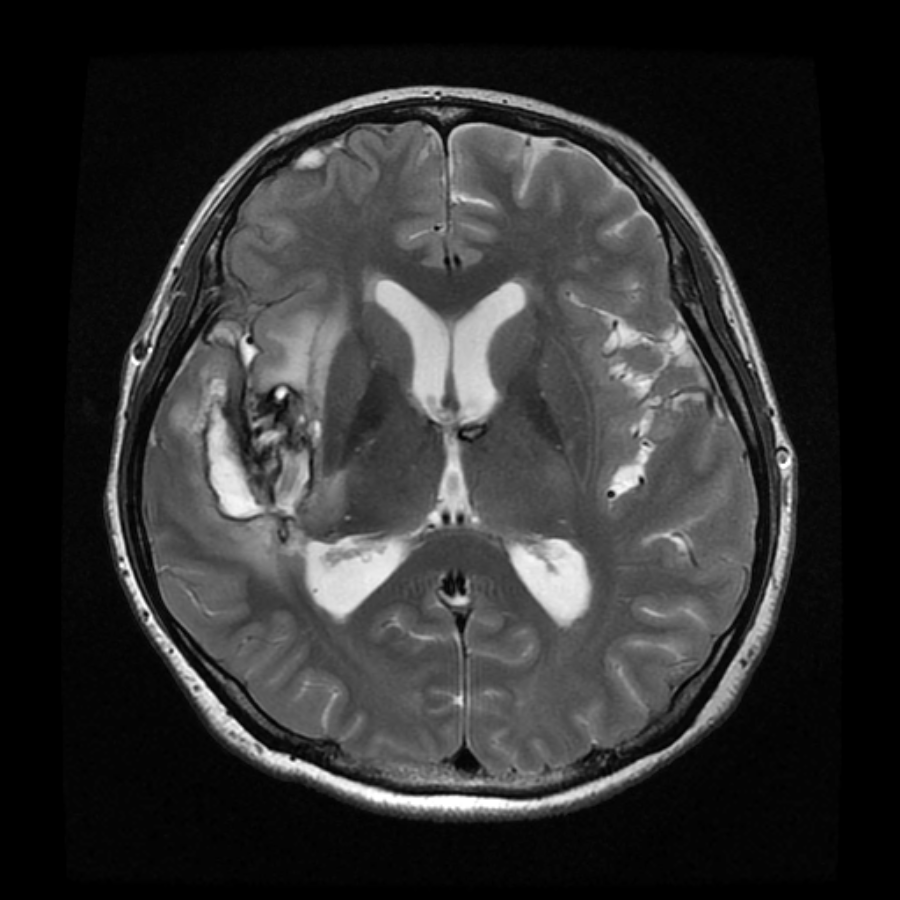 |  |
| Lesion | Lt. MCA infarction | Lt. thalamus hemorrhage | Rt. temporal lobe hemorrhage |  |
