# Supplementary material for: Therapeutic Effect of Repetitive Transcranial Magnetic Stimulation for Post-stroke Vascular Cognitive Impairment: A Prospective Pilot Study
Source: Front Neurol. 2022 Mar 22;13:813597. doi: 10.3389/fneur.2022.813597 (PMC8980431; doi:10.3389/fneur.2022.813597)
Supplement: Supplementary file 6 [file Table_6.DOCX]

**[ Supplementary Material ]** **rTMS treatment effect on fMRI**

| **Patient No.3** | | | | | | |
| --- | --- | --- | --- | --- | --- | --- |
| Region name | P cluster | Cluster size | Z_(E)_ | x | y | z |
| Left Supplementary Motor Area | 0.063 | 34 | 4.28 | -4 | 26 | 10 |
| Right Hippocampus | 0.063 | 48 | 4.15 | 22 | -34 | 58 |
| Right Postcentral | 0.063 | 56 | 4.10 | 36 | -30 | 24 |
| Left Medial Temporal | 0.063 | 21 | 4.10 | -48 | -56 | 34 |
| Left Postcentral | 0.063 | 83 | 4.07 | -30 | -38 | 28 |
| Right Angular | 0.063 | 27 | 3.35 | 44 | -46 | 24 |
| **Patient No.6** | | | | | | |
| Region name | P cluster | Cluster size | Z_(E)_ | x | y | z |
| Right Medial Frontal | 0.039 | 23 | 3.76 | 36 | 44 | 46 |
| Right Angular | 0.039 | 25 | 3.36 | 36 | -68 | -46 |
